# A mathematical model of glutathione metabolism

**DOI:** 10.1186/1742-4682-5-8

**Published:** 2008-04-28

**Authors:** Michael C Reed, Rachel L Thomas, Jovana Pavisic, S Jill James, Cornelia M Ulrich, H Frederik Nijhout

**Affiliations:** 1 Department of Mathematics, Duke University, Durham, NC 27708, USA; 2 Department of Biology, Duke University, Durham, NC 27708, USA; 3 Department of Pediatrics, University of Arkansas for Medical Sciences, Little Rock, AK 72205, USA; 4 Fred Hutchinson Cancer Research Center, Seattle, WA 98109-1024, USA

## Abstract

**Background:**

Glutathione (GSH) plays an important role in anti-oxidant defense and detoxification reactions. It is primarily synthesized in the liver by the transsulfuration pathway and exported to provide precursors for in situ GSH synthesis by other tissues. Deficits in glutathione have been implicated in aging and a host of diseases including Alzheimer's disease, Parkinson's disease, cardiovascular disease, cancer, Down syndrome and autism.

**Approach:**

We explore the properties of glutathione metabolism in the liver by experimenting with a mathematical model of one-carbon metabolism, the transsulfuration pathway, and glutathione synthesis, transport, and breakdown. The model is based on known properties of the enzymes and the regulation of those enzymes by oxidative stress. We explore the half-life of glutathione, the regulation of glutathione synthesis, and its sensitivity to fluctuations in amino acid input. We use the model to simulate the metabolic profiles previously observed in Down syndrome and autism and compare the model results to clinical data.

**Conclusion:**

We show that the glutathione pools in hepatic cells and in the blood are quite insensitive to fluctuations in amino acid input and offer an explanation based on model predictions. In contrast, we show that hepatic glutathione pools are highly sensitive to the level of oxidative stress. The model shows that overexpression of genes on chromosome 21 and an increase in oxidative stress can explain the metabolic profile of Down syndrome. The model also correctly simulates the metabolic profile of autism when oxidative stress is substantially increased and the adenosine concentration is raised. Finally, we discuss how individual variation arises and its consequences for one-carbon and glutathione metabolism.

## Background

Glutathione is a low molecular weight tri-peptide (γ-glutamyl-cysteinyl-glycine) found at relatively high concentrations (0.5–10 mM) in all mammalian cells and relatively low concentrations (2–20 μM) in plasma [1]. Inside cells, most of the glutathione (85–90%) is in the cytosol where it primarily exists in a reduced form (GSH) and to a much lesser extent as an oxidized disulfide form (GSSG). The high GSH/GSSG ratio provides the essential reducing environment inside the cell. GSH is manufactured in the cytosol by a two-step process: the first step, which combines cysteine and glutamate, is catalyzed by γ-glutamylcysteine synthetase (GCS); the second step, which adds the glycine residue, is catalyzed by glutathione synthetase (GS). Glycine and glutamate are produced and used by many metabolic reactions and have relatively high cytosolic concentrations. Cytosolic cysteine is the limiting amino acid for GSH synthesis because it has a low concentration compared to glycine and glutamate. Cytosolic cysteine comes from only three sources: (1) from methionine via the methionine cycle and the transsulfuration pathway, (2) direct import into the cell from the plasma, and (3) from excess protein catabolism over protein synthesis. Thus the availability of cysteine and the activity of GCS are the major determinants of GSH synthesis. The enzyme cystathionine-β-synthase (CBS) that catalyzes the first step in the transsulfuration pathway is highly expressed in liver cells but not highly expressed in peripheral cells, so it is not surprising that the liver is the major producer of GSH, much of which is exported to the plasma and enzymatically broken down to cysteinylglycine and cyst(e)ine that is subsequently taken up by other cells for GSH synthesis.

Glutathione is involved in many pathways that are essential for normal intracellular homeostasis. It detoxifies xenobiotics and heavy metals through a reaction catalyzed by GSH S-transferases that bind them to the sulfhydryl group on the cysteine residue. GSH plays a role in regulating lipid, glucose, and amino acid metabolism because it is necessary for the hepatic response to insulin-sensitizing agents [2]. GSH is necessary for the interconversion of prostaglandins [3]. The removal of formaldehyde, a carcinogen and a product of one-carbon metabolism, requires glutathione, and glutathione is involved in T-lymphocyte activation and viral resistance [4]. Finally, glutathione scavenges reactive oxygen species including superoxide and hydrogen peroxide. In these reactions GSH is oxidized to GSSG and the ratio [GSH]/[GSSG], an indicator of the redox status of the cell, is known to regulate redox sensitive enzymes in the pathways for cell proliferation and cell apoptosis [5]. Thus, it is not surprising that GSH (or the [GSH]/[GSSG] ratio) plays a key role in many diseases including cancer, inflammation, Alzheimer's disease, Parkinson's disease, sickle cell anemia, liver disease, cystic fibrosis, AIDS, heart attack, stroke, and diabetes [4,6] as well as in aging [7-9]. Reactive oxygen species also cause birth defects in rats, which are prevented by administration of GSH [10]. For more on glutathione chemistry and health effects, see [1,4,11-15].

**Figure 1:**
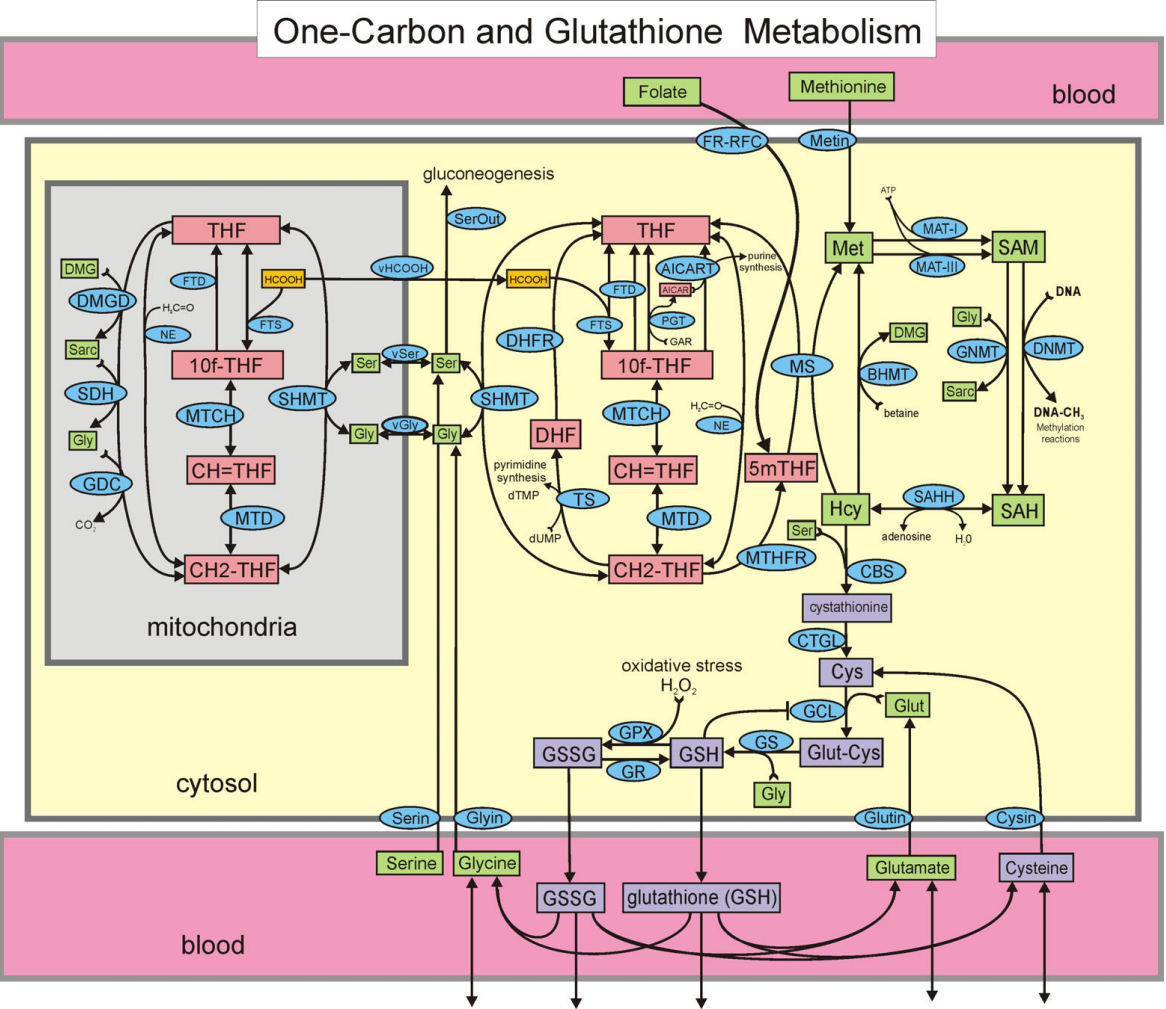
**One-carbon metabolism and the transsulfuration pathway**. Rectangles enclose the names or acronyms of substrates that are variables in the model. Substrates not in rectangles are held constant or are the products of reactions that we do not keep track of. Arrows at the bottom of the figure represent import from the gut and other cells, and losses to other cells and to degradation. There is one differential equation for each substrate. The ellipses contain the acronyms of the enzymes that catalyze the reactions. Full names for all the enzymes and substrates, as well as a complete description of the mathematical model and the values of all parameters are given in the online Additional File 1.

During the past several years we have created mathematical models for different parts of one-carbon metabolism [16-21]. The purpose of the modeling was to answer questions posed by experiments or experimentalists and to investigate mechanisms of regulation in one-carbon metabolism. In this paper, we extend our most recent model [20] to include cysteine and glutathione metabolism (Figure 1). Since this mathematical model is quite complicated, it is useful to be clear why our model needs to include all of one carbon metabolism and not just the transsulfuration pathway. First, methionine is a major hepatic source of cysteine through the methionine cycle and the transsulfuration pathway. Secondly, the redox status of the cell affects many of the enzymes in one-carbon metabolism including MATI, MATIII, MS, BHMT, as well as CBS and GCS in the transsulfuration pathway, and therefore one cannot evaluate GSH metabolism without including the methionine and folate cycles. Thirdly, patients with Down syndrome or autism have increased oxidative stress and exhibit particular disturbed profiles of one-carbon metabolism [13-15]. We would like to understand how oxidative stress (and the chromosome 21 trisomy in the case of Down syndrome) could create these disturbed profiles.

## Model Overview

Figure 1 shows the biochemical pathways in the hepatic cellular model used in this paper. Rectangular boxes represent the substrates that can vary in the model, and the ellipses contain the acronyms of the enzymes that catalyze particular reactions. There is one differential equation for each substrate that says that the rate of change of the concentration of the substrate is the sum of the reaction velocities (μM/hr) that produce it minus the sum of the reaction velocities that use it. Full names for all the enzymes and substrates are given in Additional File 1. Non-boxed substrates are taken to be constant or indicate products of reactions. This model extends the model for one carbon metabolism in [20] by adding the transsulfuration pathway, the synthesis of glutathione, and its transport into the blood. Here we discuss the main ideas involved in modeling the transsulfuration pathway, referring the reader to [20] (and its online supplementary material) for a discussion of the other parts of the model. A complete description of the full mathematical model and the values of all parameters are given in the online Additional File 1.

One of the most interesting features of one carbon metabolism is that reaction velocities are often affected not just by substrate and product concentrations and enzyme activities but also by the concentrations of substrates in distant parts of the reaction network that act as allosteric activators or inhibitors of the enzyme. As a result, the formulas for reaction velocities are often complicated functions of many variables. This is well illustrated by the velocity equation for the CBS reaction, the first step in the transsulfuration pathway:

VCBS=(Vmax[Hcy][Ser](Kmhcy+[Hcy])(Kmser+[Ser]))⋅((1.2)C([SAM]+[SAH])2302+([SAM]+[SAH])2)(ka+[H2O2]ka+[H2O2]norm)

The first factor is simply Michaelis-Menten kinetics for the CBS reaction that uses homocysteine (Hcy) and serine (Ser) as substrates. We take the Michaelis constants from the literature. The second term is the activation of CBS by SAM and SAH that was discovered by Finkelstein and Martin [22]. The form of the activation was derived by nonlinear regression on the data in [23,24]. The constant C is chosen so that the second term equals one in the normal steady state (see below). The last term is the activation of CBS by oxidative stress [25,26], which is represented by the concentration of H_2_O_2._

The differential equation for the concentration of cytosolic cysteine (Cys) is straightforward:

$$\begin{array}{l} \frac{d}{dt}[Cys]=-V_{GCS}([Cys],[Glu],[GSH],[H_{2}O_{2}])\\ +V_{CGTL}([Cysta])+V_{cysin}([bCys])-\left(\frac{(0.35){\left[Cys\right]}^{2}}{200}\right) \end{array}$$

The first term on the right is the production of cysteine from cystathionine (Cysta) by CGTL and the second term is the import of cysteine into the cell, which depends on the concentration of cysteine in the blood ([bCys]). The third term is the loss of cysteine in the reaction catalyzed by GCS that makes glutamyl-cysteine and the fourth term represents the loss of cysteine to other pathways (for example to sulfate and taurine). Cysteine also used for protein synthesis and is produced by protein catabolism; in the model we assume that these two rates balance. The form for the fourth term was chosen because the data in [27] indicate that at normal cysteine concentrations (approximately 200 μM) most of the flux away from cysteine is toward GSH and only a moderate amount towards other pathways. However, as [Cys] rises an increasing fraction is sent towards the synthesis of taurine. Formulas for V_CGTL _and V_bCYSc _(the transport of cysteine into the cell from the blood) appear in Additional File 1.

The first step in the synthesis of GSH is the formation of γ-glutamyl-cysteine (GluCys) from the constituent amino acids glutamate and cysteine by the enzyme γ-glutamyl-cysteine synthetase (GCS, also called glutamate cysteine ligase (GCL)). The reaction is reversible and GSH is a competitive inhibitor of GCS against glutamate [28-30].

The third factor in the following formula is the activation of GCS by H_2_O_2 _[25,26].

$$\begin{array}{l} V_{GCS}\;=\left(\frac{k_{a}+[H_{2}O_{2}]}{k_{a}+{\left[H_{2}O_{2}\right]}_{ss}}\right)\cdot \\ \;\left(\frac{V_{max}([Glu][Cys]-[GluCys]/K_{e})}{K_{m}^{cys}K_{m}^{glu}+K_{m}^{cys}[Glu]+K_{m}^{glu}[Gys](1+\frac{\left[GSH\right]}{K_{i}}+\frac{\left[Glu\right]}{K_{m}^{glu}})+\frac{\left[GluCys\right]}{K_{p}}+\frac{\left[GSH\right]}{K_{i}})}\right). \end{array}$$

The second step in the synthesis of GSH is the addition of the glycine residue to GluCys by the enzyme glutathione synthase (GS). We follow [30,31] and use a reversible bi-reactant Michaelis-Menten mechanism.

$$V_{GS}\;=\;\left(\frac{V_{max}([GluCys][Gly]-[GSH]/K_{e})}{K_{m}^{glucys}K_{m}^{gly}+K_{m}^{gly}[GluCys]+K_{m}^{glucys}[Gly](1+\frac{\left[GluCys\right]}{K_{m}^{glucys}})+\frac{\left[GSH\right]}{K_{p}})}\right).$$

The differential equation for cytosolic GSH is:

$$\begin{array}{c} \frac{d}{dt}[GSH]=V_{GS}([Gly],[GluCys]-V_{gsh\_out}([GSH])\\ -2V_{GPX}([GSH],[H_{2}O_{2}])\\ +2V_{GR}([GSSG],[NADPH])-(0.002)[GSH]. \end{array}$$

The first term is the synthesis of GSH from glycine and glutamyl-cysteine. The second term is the transport of GSH out of the liver cell into the blood. V_gsh_out _is actually the sum of two terms, one for the high affinity transporter and one for the low affinity transporter [32]. We ignore the canalicular transport into the bile because it is a relatively small percentage of total export [33]. The third term is the rate of production of oxidized GSSG from GSH via the enzyme GPX and the fourth term is the conversion of GSSG back to GSH via the enzyme GR. We ignore other reactive oxygen species besides H_2_O_2_, or, put a different way, H_2_O_2 _represents them all. The number two occurs in both these terms because two molecules of GSH combine to make one GSSG. In the fifth term we are assuming that 0.2% of the GSH is removed each hour in detoxification reactions that form conjugates [12].

The kinetics of sinusoidal efflux of GSH has been well studied in the perfused rat liver. The major part of the flux is carried by the low affinity transporter, which has sigmoidal kinetics with a V_max _in the range 900–1400 μM/hr, a K_m _of approximately 3000 μM, and a Hill coefficient of approximately 3 [34-36]. In our model we use a V_max _of 1100 μM/hr, a K_m _of 3000 μM, and a Hill coefficient of 3. We use standard Michaelis-Menten kinetics for the high affinity GSH transporter and for the two GSSG transporters.

We track five variables in the blood, [GSH], [GSSG], [Gly], [Cys] and [Glut]. Glycine, glutamate, and cysteine enter blood from intestinal absorption at rates that we vary in various experiments with the model; the normal rates are 630 μM/hr, 273 μM/hr and 70 μM/hr, respectively. We assume for convenience that the volume of the blood is the same as the volume of the liver. Glycine, cysteine, and glutamate leave the blood by transport into liver cells (depending on their concentrations) and they are also formed in the blood by the breakdown of GSH and GSSG into their component amino acids [37,38]. We also assume that normally 10% of the cysteine, glycine, and glutamate, in the blood is taken up per hour by other cells and that an additional 25% of cysteine is converted to cystine. Under normal conditions a large percentage of blood GSH and GSSG is broken down into the component amino acids and a small amount is taken up by other cells or otherwise leaves the system. As above, full details and formulas appear in Addition File 1.

For each *in silico *computation, the values of various constants (like H_2_O_2_) are given, as are the methionine and serine levels in the blood, and the rates of input of cysteine glutamate, and glycine into the blood. These are the "inputs" to the model. The differential equations are then solved to determine the steady-state values of the concentrations of all the variables and the steady state rates of all the reactions. Of course, if the inputs are different the steady state will be different. We experiment with the model by changing the inputs or changing parameters (for example, a parameter that gives the strength of a particular allosteric interaction) and determine what the effect is. By removing interactions we can take the model apart piece by piece so that we can understand how and why glutathione metabolism works the way it does. We also allow the inputs to vary as functions of time (for example the amino acid input will vary because of meals) and compute the time course of each concentration and reaction rate. This allows us to investigate the homeostatic mechanisms that protect the system against fluctuations in the inputs.

A number of substrate concentrations are fixed in the model and in all the simulations reported below. These include: cytosolic GAR (10), NADPH (50), betaine (50), formaldehyde (500), dUMP (20), and total cellular folate (20). All concentrations are in μM.

### Limitations of the model

This model was designed to allow us to study various regulatory mechanisms in the transsulfuration pathway and the effects of oxidative stress, particularly as applied to Down syndrome and autism. No mathematical model can track all of the variables that might affect a complex biochemical system such as glutathione metabolism. This is also true, of course, in biological experimentation. This model is no exception. We ignore canalicular excretion of GSH. We use K_m _values in the ranges determined experimentally but there is much less information on V_max _values. Often we choose V_max _values so that the steady state concentrations of substrates and products lie within the normal published ranges. Cellular amino acid concentrations are increased by feeding and protein degradation and decreased by protein synthesis, growth and use in one-carbon metabolism. In this model we assume that protein synthesis and degradation are in balance and that no amino acids are used for growth. The consequences of this assumption are outlined in the discussion.

One-carbon metabolism and the transsulfuration pathway contain many allosteric interactions by which substrates in one part of the pathway affect the activity of distant enzymes. We use experimentally determined forms for these allosteric interactions but sometimes the details of the kinetics are not known, forcing us to make reasonable educated guesses. Similarly, many effects of oxidative stress on the enzymes of one carbon metabolism and the transsulfuration pathways are known but detailed kinetics are not available.

In this paper we are mainly interested in intracellular liver metabolism, so we take a somewhat simple view of the fates glutathione and its metabolites in the blood. Future work will include a more detailed model of the blood compartment and inter-organ regulation of glutathione and its component amino acids. Thus, we do not expect that our model will make perfect quantitative predictions. Rather, we want to use it to investigate the qualitative features of glutathione metabolism in the normal state and in various disease states.

## Results

### A. Normal model steady-state concentrations and velocities

We take the normal values of inputs to be the following. Blood methionine is 30 μM and blood serine is 150 μM. The rates of cysteine, glycine, and glutamate input to the blood are 70 μM/hr, 630 μM/hr, and 273 μM/hr respectively. The normal concentration of H_2_O_2 _is 0.01 μM. With these inputs, the model computes the concentrations of the cytosolic variables given in Table 1.

**Table 1:** Normal model cytosolic concentrations (μM)

Folates	Methionine cycle	Transsulfuration	Other
THF = 4.61	Met = 49.2	Cysta = 36.9	Ser = 563
510CH = 0.28	SAM = 81.1	Cys = 195	Gly = 924
510CH2 = 0.51	SAH = 19.1	GluCys = 9.8	Sarc = 9.16
10fTHF = 3.41	Hcy = 1.12	GSH = 6591	DMG = 0.71
5mTHF = 4.50		GSSG = 61.3	Aicart = 0.94
DHF = 0.039		Glut = 3219	HCOOH = 13.1

These model values correspond well to the values in the literature. For cytosolic folate variables and methionine cycle variables, see the discussions in our previous papers [16-21]. Typical values of GSH in animal cells are in the range 500–10,000 μM or 0.5–10 mM [1,39]. Typical values for cysteine are 150–250 μM [1] and for cystathionine 40 μM [40]. The ratio [GSH]/[GSSG] is thought to be around 100 for cells that are not under oxidative stress [41], and [GSH]/[GSSG] = 107.5 in our model cell.

The computed velocities of the cytosolic reactions are given in Table 2. There is very little information in the literature about reaction velocities because they are difficult to measure. However, the model concentration of GSH declines in the fasting state about as rapidly as observed experimentally (See Section B, below). This indicates that the overall rates of GSH production from cysteine and methionine and the transport of GSH out of the cell are in the appropriate ranges. We also note that the flux around the methionine cycle is 205 μM/hr and approximately half enters the transsulfuration pathway (V_CBS _= 103 μM/hr) and half is remethylated to methionine in accordance with the results of Finkelstein and Martin [22].

**Table 2:** Normal model cytosolic reaction velocities (μM/hr)

Folate Cycle	Methionine cycle	Transsulfuration
V_MTD _= -103	V_MATI _= 125	V_CBS _= 103
V_MTCH _= -103	V_MATIII _= 80.5	V_CTGL _= 103
V_FTS _= 552	V_GNMT _= 61.6	V_GCS _= 1250
V_FTD _= 72.8	V_DNMT _= 144	V_GS _= 1250
V_ART _= 188	V_SAHH _= 205	V_GPX _= 312
V_PGT _= 188	V_MS _= 40.2	V_GR _= 269
V_SHMT _= 12.1	V_BHMT _= 61.9	
V_NE _= 58.6		
V_TS _= 133		
V_DHFR _= 133		
V_MTHFR _= 40.3		

The computed concentrations of variables in the blood are given in Table 3. Wu et al. report that the combined cysteine and cystine concentrations are 110–325 μM [1]. In our model the computed plasma cysteine concentration is 186 μM, which is in the middle of this range. Plasma concentrations in humans are reported in the range 2–20 μM for GSH [1,13,14], and 0.14–0.34 μM for GSSG [13,14,42]. The average plasma GSH/GSSG ratio is reported to be in the range 25–28 μM with a large standard deviation [14,15], and in the model it is 26.5. Plasma glycine levels are reported to be approximately 300 μM in [43].

**Table 3:** Normal model blood concentrations (μM)

Cys = 186	Gly = 221	Glut = 60.4	GSH = 12.7	GSSG = 0.48

The computed values of various transport rates are given in Table 4. We use the abbreviations o = outside, b = blood, c = cytosol, so, for example, V_oCysb _is the transport of cysteine from the outside into the blood. V_oCysb_, V_oGlyb_, and V_oGlutb _are inputs to the model. All other transport velocities are computed by the model. The second row shows the transport velocities of the five amino acids in the model from the blood into liver cells. The third row shows the transport velocities of GSH and GSSG from the cell into the blood. Detailed kinetic information is available on amino acid transporters [44,45] and on the high and low affinity transporters of GSH and GSSG [32,39,46] and we chose our kinetics parameters from this literature.

**Table 4:** Normal model net transport velocities (μM/hr)

V_oCysb _= 70.0	V_oGlyb _= 630.0	V_oGlutb _= 273		
V_bCysc _= 1213	V_bGlyc _= 1816	V_bMetc _= 103	V_bSerc _= 787	V_bGlutc _= 1475
V_cGSHb _= 1152	V_cGSSGb _= 36.3			
V_bGSHo _= 8.9	V_bGSSGo _= 3.6	V_bCyso _= 64.9	V_bGlyo _= 22.1	V_bGluto _= 6.0

The fourth row in Table 4 requires more comment. Our main interest is to understand the synthesis and export of GSH in liver cells and how intracellular metabolite balance is affected by oxidative stress. Since GSH is exported rapidly from liver cells and much of the export is broken down into the constituent amino acids that are then reimported into liver cells, it was necessary to include the blood compartment in our model. The blood communicates with all other tissues none of which are in our model. We have therefore necessarily made a number of assumptions about the loss of GSH, GSSG, Cys, Gly, and Glu to other tissues. For example, as discussed above, we assume that normally 10% per hour of the cysteine, glycine, and glutamate in the blood is taken up by other cells and that an additional 25% of cysteine in the blood is lost by conversion to cystine. The velocities in the fourth row reflect these assumptions.

### B. The Half-life of Glutathione

Ookhtens et al. [34] reported that when buthionine sulfoximine is used to inhibit the activity of GCS (which catalyzes the first step in GSH synthesis) a half life of 2–6 hours for cellular GSH is observed. This is consistent with the experiments of [47]. Moreover, the rate of sinusoidal GSH efflux in both fed and starved rats is near saturation at about 80% of Vmax, about 1000–1200 μM/h [34]. Thus, if the cytosolic GSH concentration is approximately 7000 μM, then the half life would be in the 2–3 hour range. Therefore, a variety of experimental studies and calculations consistently suggest a short half life in the 2–3 hour range.

By contrast, Aw et al. [33] report that rats fasted for 48 hours lose approximately 44% of the intracellular GSH in their hepatocytes. They also report that after 48 hours the rate of GSH transport out of the cell declined by 38%. These results are consistent with Tateishi et al. [48,49] who reported a decline in liver GSH to a level between one half and two thirds of normal after a 48 hour fast. These experiments suggest a half-life longer than two days. One possible explanation for this long half-life under starved conditions is that the normal dietary amino acid input is partly replaced by protein catabolism. However, given the normal rate of GSH efflux, a 48 hour half-life would require that catabolism replace 94% of daily dietary input, which seems improbably high.

**Figure 2:**
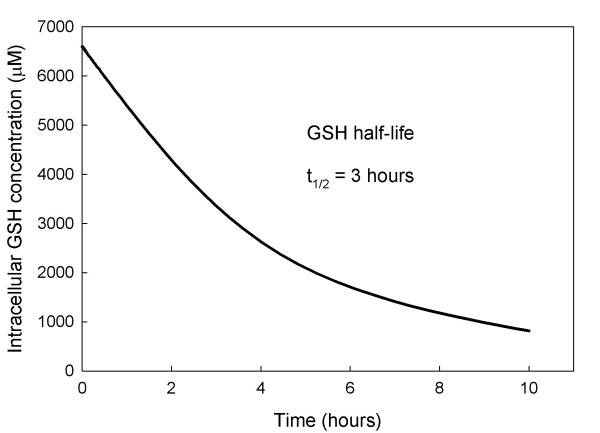
**GSH half life after GCS is blocked**. When GSH synthesis is stopped the model intracellular GSH concentration declines rapidly with a half life of approximately 3 hours.

An alternative explanation, which could potentially explain both sets of experiments, is that exported GSH is broken down into constituent amino acids in the blood that are rapidly reimported into the liver cells. Indeed, it is known that the enzyme γ-glutamyltranspeptidase (GGT) on the external cell membrane initiates this process (called the γ-glutamyl cycle) [12,50,51]. In our model the computed value of GSH transport out of the cell (Table 4) is V_cGSHb _= 1152 and the rates of Cys, Gly, and Glut import are also high (Table 4), although we assume that 10% per hour of the amino acids in the blood are lost to non-liver cells and an additional 25% of Cys is lost by conversion to cystine. Figure 2 shows the cytosolic concentration of GSH in our model liver cells for 10 hours after the concentration of the enzyme GCS was set to zero. The computed half life of GSH is 3 hours.

**Figure 3:**
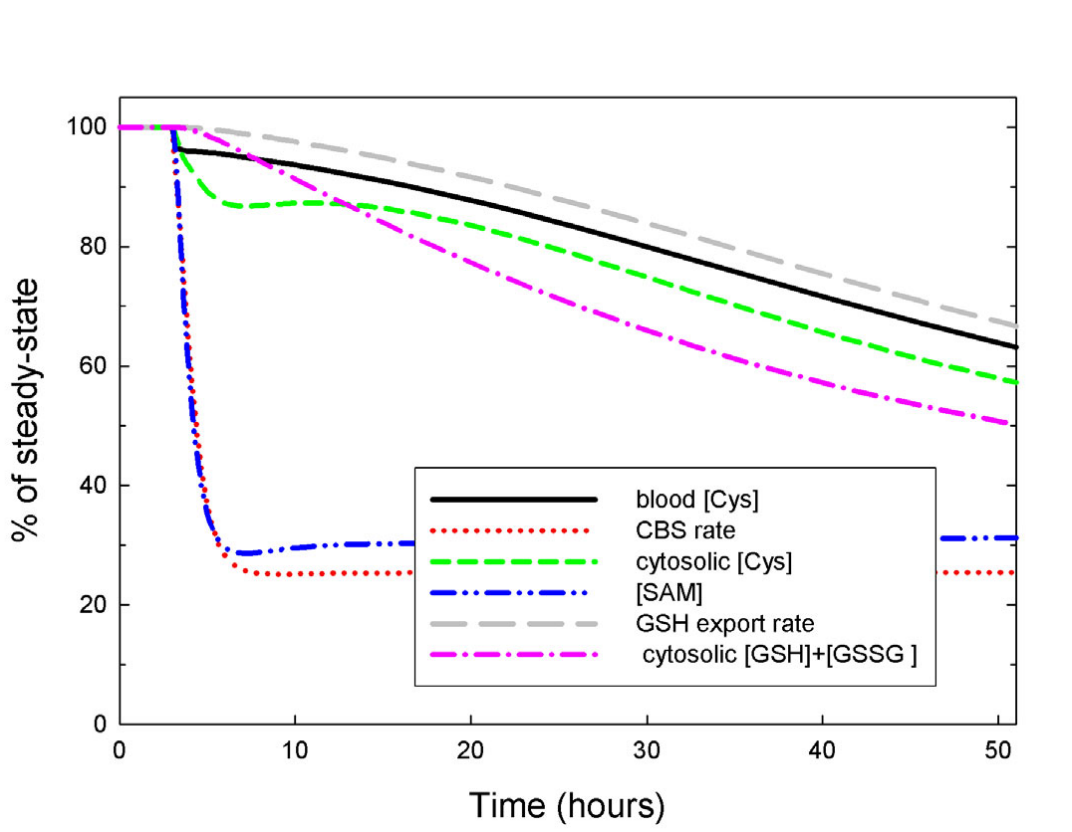
**GSH and GSH transport under fasting conditions**. After three hours, the inputs of cysteine, methionine, glycine, glutamate, and serine are reduced to 1/3 of normal. The intracellular GSH + GSSG concentration declines slowly over the 48 hour period to about 50% of normal and the rate of GSH export declines to 67% of normal consistent with the experiments reported in [33]. The cytosolic and blood cysteine concentrations decline proportionally to GSH. The methionine cycle metabolites and fluxes equilibrate rapidly.

Figure 3 shows the concentration of GSH and other metabolites in our model liver cell during a fasting experiment over a 48 hour period. We assume that during fasting, protein catabolism supplies 1/3 of the normal amino acid input. The GSH concentration declines slowly over the 48 hour period to about 50% of normal and the rate of GSH export declines to 67% of normal consistent with the experiments reported in [33]. Thus the rapid reimport hypothesis explains both sets of data. Other metabolites show interesting changes during the fast. The methionine cycle metabolites adjust very rapidly to the decreased methionine input reaching new steady states within a few hours. However, the metabolites in the GSH synthesis, export and reimport pathway decline very slowly, achieving their new steady states in 4–5 days (data not shown).

Mosharov et al. [26] studied the role of the transsulfuration pathway in GSH synthesis. When they blocked CTGL they observed that that the intracellular GSH concentration dropped to a new steady state of approximately 42% of control in about 24 hours. They concluded that approximately half of GSH is derived via the transsulfuration pathway. Our methionine input is computed to be 103 μM/h consistent with experimental measurements [26,52] and our cysteine input into the system is 70 μM/h. If we remove the methionine input in the model, the GSH concentration declines to a new steady-state 47% of normal. On the other hand, if we remove cysteine input the GSH concentration declines to a new steady-state 68% of normal. These model results support the interpretation in [26] that methionine and cysteine inputs contribute equivalently to GSH synthesis. We remark that one would not expect the contributions of the two metabolites to GSH synthesis to be strictly proportional to their inputs since they are used in other reactions and the system is highly non-linear.

### C. Inhibition of GCS

**Figure 4:**
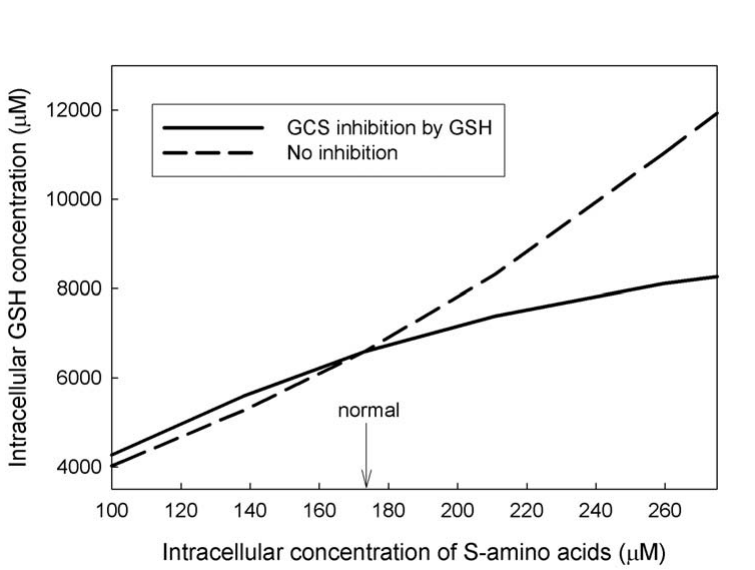
**Effect of GSH feedback inhibition**. The GSH concentration is plotted as a function of the intracellular concentration of sulfur amino acids. The solid curve shows the GSH concentration when the inhibition of GCS is included in the model. The dashed curve shows the GSH concentration when the inhibition by GSH is removed.

It is well known [27,41] that GSH is a competitive inhibitor against glutamate of GCS, the enzyme that catalyzes the synthesis of glutamyl-cysteine. This inhibition can naturally be thought of as product inhibition, one step removed. As glutathione rises it indirectly inhibits its own synthesis and as glutathione falls the inhibition is released. Figure 4 shows that this inhibition has the effect that is expected at steady state. As sulfur amino acid input rises, so does glutathione concentration but not as fast as it would if the inhibition were not present. At low sulfur amino acid concentrations the effect is small. Thus the primary effect of the inhibition is to prevent excess accumulation of GSH. Such accumulation would sequester more amino acids, would increase transport out of the cell up to saturation, and would therefore increase the loss of cysteine to cystine in the blood.

### D. Stability of GSH under large input fluctuations

**Figure 5:**
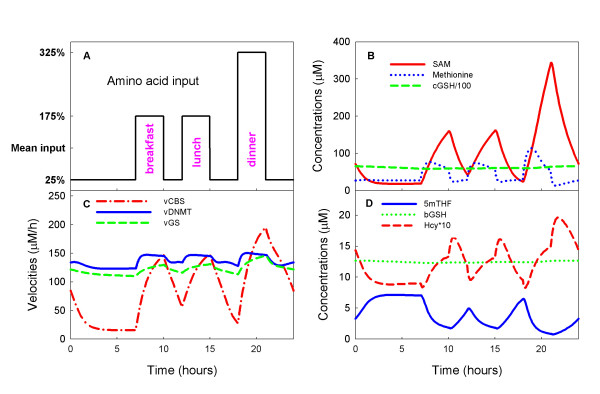
**The Stability of the glutathione pools in the face of input fluctuations**. Panel A shows the amino acid input to the model hepatocytes throughout a 24 hour day. The input is 25% of the mean while fasting, 175% of the mean for three hours after breakfast and lunch, and 325% of the mean for three hours after dinner. For discussion, see the text. Panel B shows moderate variations in methionine concentrations and extremely large swings in SAM concentration, but GSH concentration remains stable. Panel C shows that the velocity of the CBS reaction varies dramatically, but the velocity of the GS reaction, which synthesizes glutathione, shows milder variation. As expected, the long range allosteric reactions between the folate cycle and the methionine cycle stabilize the velocity of the DNA methylation reaction (vDNMT). Panel D shows that there are large variations in 5mTHF and Hcy throughout the day, but the GSH concentration in the blood remains stable.

Hepatocytes are seldom at steady state [21] because they receive large protein inputs during and shortly after meals and relatively little protein input between meals. How do these daily fluctuations affect the intracellular and blood glutathione pools? To investigate this question, we varied the amino acid input (cysteine, glycine, glutamate, methionine, and serine) throughout the day and used the model to compute the time courses of all the concentrations in the model, and all the velocities. Let A denote the daily average input of a particular amino acid. While fasting (for example, from 12 midnight until 7 am), we assume that the input is (0.25)A. From 7 am to 10 am, we assume that the input is (1.75)A corresponding to breakfast, followed by two hours of fasting. Then, from 12 noon until 3 pm we assume that the input is (1.75)A corresponding to lunch, followed by three hours of fasting and then an input of (3.25)A for three hours corresponding to dinner. The complete daily input profile is shown in Figure 5A.

Panel B of Figure 5 shows that the fluctuations in methionine input cause very large fluctuations in SAM but only modest fluctuations in intracellular methionine concentration, as suggested by the early methionine loading experiments of Finkelstein and co-workers [53,54]. Corrales et al. [55] have suggested that the relative stability of methionine is due to the complicated kinetics of MAT-I and MAT-III and we have confirmed this by model experiments [56]. Panel B also shows that the intracellular GSH concentration (divided by 100 for graphing purposes) also remains stable throughout the day.

Panel C of Figure 5 shows that the velocity of the CBS reaction tracks the methionine input as expected, but that the velocity of the GS reaction by which GSH is synthesized has milder fluctuations. These smaller fluctuations are a result of the inhibition of the GCS reaction by GSH (see Section C). In addition, the intracellular GSH pool is normally very large (6591 μM in our model, see Table 1). Both of these effects contribute to the impressive stability of the GSH concentration, seen in Panel B, in the face of large fluctuations in amino acid input. Note that the velocity of the DNA methylation reaction (DNMT in Panel C) is also quite stable despite the fact that its substrate, SAM, is undergoing very large fluctuations. It is understood that this is a result of long-range allosteric interactions between the methionine cycle and the folate cycle [18].

Panel D of Figure 5 shows that homocysteine undergoes large fluctuations in synchrony with the methionine input as expected. 5mTHF also fluctuates but in the opposite direction from homocysteine for two reasons. First, when methionine input rises dramatically, so does [SAM] and SAM inhibits MTHFR. Secondly, when homocysteine rises, it drives the MS reaction faster, which reduces 5mTHF. Finally, the blood concentration of GSH remains completely stable despite the large transient amino acid input fluctuations.

### E. Oxidative Stress

**Figure 6:**
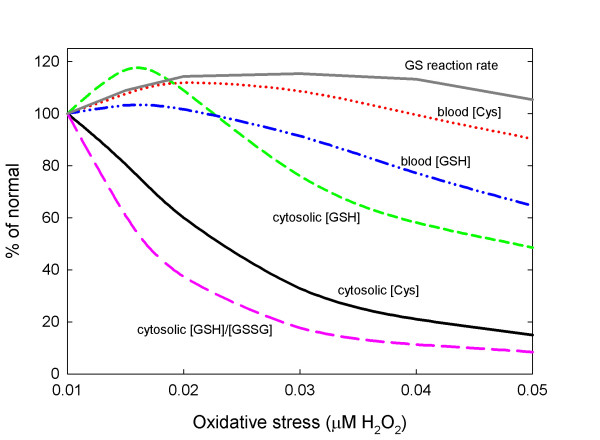
**Effect of oxidative stress**. The curves show the effect on the steady-state values of cysteine and GSH in the cytosol and the blood, the rate of GSH synthesis by GS, and the cytosolic [GSH]/[GSSG] ratio as H_2_O_2 _concentration is raised from normal (0.01 μM) to 0.05 μM.

In our model, oxidative stress is represented by the concentration of H_2_O_2_. When H_2_O_2 _increases there are several effects on one-carbon metabolism. First, the increased concentration of H_2_O_2 _inhibits the enzymes MS and BHMT and activates the enzymes CBS and GCS [25,26]. Secondly, the balance of GSH and GSSG is shifted toward GSSG via the GPX and GR reactions. This affects the upstream metabolites in the methionine and transsulfuration pathways because GSSG inhibits the enzymes MAT-I and MAT-III [57,58]. All of these influences are in the model; for details, see Additional File 1. The response to oxidative stress in the model is surprisingly complex; see Figure 6.

Under moderate oxidative stress there are moderate increases on blood and cytosolic GSH and blood cysteine, while cytosolic cysteine and the [GSH]/[GSSG] ratio decline. Cytosolic GSH increases because oxidative stress activates CBS and GCS increasing the flux through the GCS and GS reactions and simultaneously lowering the cytosolic cysteine concentration. Since cytosolic GSH increases, the export out of the cell will also increase thus raising the blood GSH and blood cysteine concentrations. The elevated H_2_O_2 _concentration drives the balance in the GPX and GR reactions towards GSSG thus lowering the [GSH]/[GSSG] ratio. Under high oxidative stress this balance is shifted even further towards GSSG and this has consequences for overall cysteine balance.

In the model cytosolic GSH has three fates: it is transported into the blood, there is a net flux to GSSG, and 0.2% is removed per hour corresponding to detoxification reactions and excretion into the bile. Likewise, cytosolic GSSG has two fates: it is transported into the blood, and 10% is removed per hour corresponding to excretion into the bile. Of course, removal of one GSH or GSSG results in the removal of one or two cysteines, respectively. At normal steady state concentrations the cysteine lost by these two mechanisms are about equal. However, as the oxidative stress increases and the balance between GSH and GSSG shifts toward GSSG, more cysteines are lost from the system per hour. At moderate oxidative stress this effect small. However, with high or chronic levels of oxidative stress this effect gets much larger and the loss of cysteines is quite large. This causes the rate of the GS reaction to come back down to normal despite the upregulation of CBS and GCS and cause the steady concentrations of cytosolic GSH and the blood concentrations of GSH and cysteine to decline below normal; see Figure 6.

### E. The Metabolic Profile of Down Syndrome

Down syndrome is a complex metabolic and genetic disorder whose root cause, trisomy 21, is an extra copy of chromosome 21 [59]. Down syndrome is not rare; it occurs in approximately 1 out every 700–800 live births [60]. Children with Down syndrome have abnormal metabolic profiles and show increased incidence of a large number of serious diseases including leukemia and diabetes [61]. In most cases, it is not understood whether these diseases are caused by the extra chromosome, the altered metabolic profile, or both.

To investigate the metabolite profile of Down syndrome using the model, we began by increasing by 50% the V_max _of CBS, since the gene for CBS is on chromosome 21 and is expressed at 150% of normal. The first column of Table 5 shows the average percent change in the levels of six *plasma *metabolites in 42 Down patients compared to controls (taken from [13]). The second column shows the percentage change in these metabolites in the model when the V_max _of CBS is increased by 50%. Note that the intracellular concentrations of Hcy, SAM, SAH, and Met all change in the same direction as seen clinically. We would not expect a close match to the clinically observed percentage changes because we are comparing intracellular model changes to blood measurements. The increased dosage of CBS has almost no effect on the model plasma concentrations of bCys and bGSH. Thus these changes must come from some other effect of chromosome 21 trisomy.

**Table 5:** Down syndrome: Clinical and model metabolic changes (%)*

substrate	clinical change	CBS × 1.5 H_2_O_2 _normal	CBS × 1.5 H_2_O_2 _× 2.5
Hcy	-24	-29	-35
Met	-47	-5	-15
bCys	15	0.2	13
bGSH	-14	0.2	-2
SAM	-18	-21	-48
SAH	-22	-26	-49
Cys	--	2	-55
5mTHF	--	29	117
CH2-THF	--	-14	-51
10fTHF	--	-7	-34

* Clinical data in Column 1 are % changes in blood levels of metabolites averaged over 42 Down patients compared to 38 control patients [13]. Columns 2 and 3 show % changes in model values. In columns 2 and 3, bCys and bGSH indicate model blood concentrations, and the other metabolites are intracellular. Column 2 shows the effect of increasing the dosage of CBS by a factor of 1.5. Column 3 shows the effect of increased CBS combined with moderate oxidative stress. For discussion, see the text.

It is known that Down patients suffer from mild to moderate oxidative stress due to the overexpression of the Cu-Zn superoxide dismutase (SOD) gene that is also located on chromosome 21 [62]. Column 3 in Table 5 shows the effects on metabolite concentrations when the H_2_O_2 _concentration (normally 0.01 μM) is increased to 0.025 μM in addition to the increased dosage of CBS. The methionine cycle metabolites are further reduced compounding the effects of trisomy 21. Blood cysteine increases substantially and blood GSH declines modestly. The reason for the increase in [bCys] under mild oxidative stress is discussed in Section E.

A number of clinical observations suggest that Down patients may have a functional folate deficiency despite having normal plasma levels of folate and vitamin B_12 _[13]. The model results support the discussion in [13] of why this is so. The increased expression of CBS lowers the concentration of Hcy and therefore lowers the rate of the MS reaction. Thus, folate builds up in the form of 5mTHF (the "methyl trap") and there is less folate in the forms CH_2_-THF and 10f-THF that are the substrates for thymidylate and purine synthesis, respectively. Indeed, the last three rows of Table 5 show that the up-regulation of CBS has exactly this effect and that the addition of oxidative stress makes the effect much stronger.

### F. The Metabolic Profile of Autism

Autism is a complex neurodevelopmental disorder whose cause is unknown and which is diagnosed solely on behavioral criteria [63]. The incidence of autism has been increasing and is now thought to be present in ~1 in 150 children in the United States [64]. Numerous studies suggest that both genetic and environmental influences are important in the development of autism [65-67]. Some studies have identified metabolic deficiencies in autistic children [14], and it has recently been shown that there is a characteristic metabolic profile in many autistic children involving disturbances in methionine and glutathione metabolism [14,15]. The first and second columns in Table 6 show the changes in blood levels of various metabolites in autistic children compared to normal patients in two different clinical studies [14,15].

**Table 6:** Autism: Clinical and model metabolic changes (%)*

substrate	clinical change 1	clinical change 2	H_2_O_2 _× 5	H_2_O_2 _× 5 adenosine up
Hcy	-5	-9	-19	-39
Met	-26	-39	-20	-26
bCys	-20	-19	-9	-9
bGSH	-32	-46	-35	-34
SAM	-10	-22	-61	-22
SAH	24	49	-54	24
bGSH/bGSSG	-48	-66	-88	-88
5mTHF	--	--	154	144
vDNMT	--	--	-6	-10

*Clinical data in columns 1 and 2 are average changes in blood levels of metabolites in autistic children compared to control patients [14, 15]. Columns 3 and 4 show % changes in model values. In columns 3 and 4 bCys, bGSH and bGSSG indicate model blood concentrations, and the other metabolites are intracellular. vDNMT is the velocity of the DNA methylation reaction (μM/hr). Column 3 shows the effect of severe oxidative stress that is a factor of 5 higher than normal. Column 4 shows the effect of adding increased adenosine concentration to the oxidative stress. For discussion, see the text.

One of the working hypotheses in [14,15] is that autistic children have a self-perpetuating cycle of inflammation and oxidative stress. Indeed, it is reported that autistic children have increased levels of brain and gut inflammation [68,69]. The fact that blood GSH levels in autistic children were down 32–46% and the blood GSH/GSSG ratio was down 48–66% is also consistent with this hypothesis (Table 6). Oxidative stress is represented in the model by the concentration of H_2_O_2_. The H_2_O_2 _concentration drives the redox balance in the cell from GSH toward GSSG (see Figure 1) and the increased concentrations of H_2_O_2 _and GSSG can modulate many enzymes in one-carbon metabolism (see Section E). In Column 3 of Table 6 we show the result of a substantial increase of oxidative stress in the model to 5 times the normal level. The model intracellular concentrations of Hcy, Met, and SAM all fall dramatically corresponding to the clinically observed declines in their plasma concentrations. The model concentration of bGSH and the bGSH/bGSSG ratio both decline in the plasma similarly to the degree of decline seen in the autistic patients in the clinic. Finally, we note that severe oxidative stress causes a "methyl trap" such that the model concentration of 5mTHF more than doubles. There are several underlying reasons for the methyl trap: the concentration of Hcy drops, oxidative stress inhibits MS, and the drop in [SAM] releases the inhibition of MTHFR, which drives more folate towards the 5mTHF form.

There is one apparent discrepancy between the model results in column 3 (Table 6) and the clinical results in columns 1 and 2. The model intracellular concentration of SAH declines under severe oxidative stress while in patients the average concentration of SAH in the blood went up 24–49%. It is reported in [14] that the plasma adenosine concentration was significantly elevated on the average in autistic children compared to controls, though both populations showed very large standard deviations. The observed plasma increase in adenosine could be due to a decrease in adenosine deaminase activity, inhibition of adenosine kinase, or an increase in 5-nucleotidase, all of which can occur in the presence of oxidative stress [70-72]. In our model, intracellular adenosine concentration is a constant. When we raised the constant by a factor of 5, we obtained the results in column 4 (Table 6). The metabolic profile is very similar to that in column 3 except that now the intracellular SAH concentration does not decline but instead increases by 24% in line with the clinical data.

Finally, we note that the model predicts modest decreases in the rate of the DNA methylation reaction (vDNMT) and this is consistent with the reported global DNA hypomethylation seen in autistic children [73]. The decrease in reaction rate is caused by the decrease in [SAM] and the increase in [SAH], which inhibits DNMT.

## Discussion

We have expanded our previous model of one-carbon metabolism to include the transsulfuration pathway, the synthesis and export of glutathione, and its breakdown in and export from the blood. This has enabled us to investigate a number of specific problems about glutathione metabolism from the experimental and clinical literature.

We have shown that the glutathione pool is very insensitive to the large variations inacute amino acid input caused by daily meals. In some cases we have been able to give causal explanations for observed experimental phenomena and we are able to test the quantitative effects of various allosteric inhibitions and excitations. Finally, we used to model to show how the observed metabolic profile of Down syndrome could arise from trisomy 21 and moderate oxidative stress and how the observed metabolic profile of autism patients could arise from high oxidative stress and increased adenosine concentration.

In a series of papers [34-36], Ookhtens and colleagues showed that the half-life of glutathione in the liver was quite low, approximately 2–3 hours, consistent with the result of Lauterburg [47]. On the other hand, the same group showed in [33] that glutathione has a half-life of approximately two days in the livers of fasted rats. In Section B we confirmed that there is no contradiction between these results. In the first case, GSH synthesis is blocked, so the rapid export of GSH makes the GSH concentration decline rapidly. In the second case, even though the rats are fasted, the rapid reuptake of cysteine, glycine, and glutamate by the liver cells insures that the synthesis of GSH declines relatively slowly and therefore the observed half-life is long. Finally, the model results support the conclusions of Mosharov et al. [26] that both cysteine and methionine contribute approximately equally to GSH synthesis in the liver. This is true even though GSH is exported rapidly and cysteine is reimported rapidly compared to the methionine input.

Lu proposes in [12] that the high glutathione concentration in hepatocytes is a storage mechanism for cysteine. But what is the reason for the rapid cycling, i.e. fast export of GSH, breakdown by GGT, and fast reimport of cysteine? This is a futile cycle that requires a lot of energy. A reasonable hypothesis is that the rapid cycling allows the liver to respond quickly to the glutathione requirements of other tissues. This hypothesis is consistent with the idea that glutathione is a mechanism for cysteine storage [12], but also helps explain the reason for the γ-glutamyl cycle and the reason for the short half-life of hepatic GSH.

Cell metabolism is very complicated and the same substrate is often used in many different reactions. As a result the response function of a metabolite or a reaction velocity to changes in a parameter or input may be nonlinear and non-monotone. For example, in Section E we showed that moderate oxidative stress causes blood GSH and blood cysteine to rise, but severe oxidative stress causes blood GSH and blood cysteine to fall. This increase at low oxidative stress is due to the stimulation of CBS and GCS that increases GSH synthesis and concentration, and therefore the rate of export. At high or chronic oxidative stress, however, the model suggests that the balance shifts towards GSSG, and removal of cysteine in the form of GSSG dominates, resulting in a decline in cysteine.

There is increasing evidence that oxidative stress plays a role in the development of autism [74]. The metabolic profile of autistic patients has been shown to be abnormal with elevated biomarkers that indicate chronic oxidative stress and evidence that GSH synthesis may be insufficient to maintain redox homeostasis [14,15]. Likewise, the overexpression of SOD is children with Down syndrome leads to a reduction of GSH and an increase in oxidative stress [13]. In our model oxidative stress is represented by an elevated level of H_2_O_2 _which induces many changes in one-carbon metabolism and the transsulfuration pathway. H_2_O_2 _stimulates CBS and GCS and inhibits MS and BHMT. In addition H_2_O_2 _drives the GSH/GSSG balance towards GSSG, which inhibits MAT I and MAT III. We have found that, in our model, oxidative stress alone can produce some but not all the metabolic characteristics of Down syndrome and autism. However, the addition of trisomy 21 in the first case, and raised adenosine in the second, brings the profiles much closer to those observed in patients with Down syndrome and autism, respectively.

Cellular amino acid concentrations are increased by feeding and protein degradation and decreased by protein synthesis, growth and use in one-carbon metabolism. During early growth (until about the second year after birth), about 10–20% of the amino acid pool is used in growth and is thus not available for GSH synthesis and one-carbon metabolism [75-77]. This would be expected to have an effect on the rates amino-acid-requiring processes of one-carbon metabolism and glutathione synthesis. We have found, by simulation, that if we reduce the amino acid input into the system by 15%, the concentration of GSH and the synthesis rate of GSH are proportionally diminished, but there is little effect on the DNA methylation reaction, while reactions in the folate cycle are reduced by 2–9%. This reduction in GSH synthesis may contribute to excessive oxidative stress in infants.

Calculations with the model show that blood concentrations do not necessarily reflect intracellular concentrations of metabolites. For example, the increased dosage of CBS and GCS in our simulation of Down syndrome causes the intracellular concentration of cysteine to decline while the blood concentration increases. This shows that care must be taken in interpreting blood measurements, and that ideally one would like to conduct experiments in which both intracellular and extracellular concentrations are measured. By contrast, we found in the model (simulations not shown) that the blood concentrations of GSH and GSSG track the intracellular concentrations.

The purpose of this model was to study the properties of intracellular glutathione metabolism, in particular the effects of oxidative stress and trisomy 21. Of course intracellular glutathione metabolism is affected by the import of amino acids and the export and removal of GSH and GSSG. We therefore needed include a blood compartment and to keep track of bCys, bGly, bGSH, bGSSG and bGlut. The kinetics of the contents of the blood compartment are complicated because GSH and its component amino acids in the blood interact with the kidney, the brain, and other tissues. These important interactions are beyond the scope of this initial investigation, but will be incorporated in future studies.

This model provides a tool, different from laboratory experimentation, that can be used to experiment with one-carbon and glutathione metabolism. By using the tool, it is possible to quickly and easily test hypotheses, evaluate new ideas, and give causal explanations for what is seen in the clinic and the lab. In this spirit, the authors welcome questions, suggestions, and hypotheses to test from clinicians and experimentalists.

## Abbreviations

**Enzymes**. AICART: aminoimidazolecarboxamide ribonucleotide transferase; BHMT: betaine-homocysteine methyltransferase; CBS: cystathionine β-synthase; CTGL: β-cystathionase; DHFR: dihydrofolate reductase; DMGD: dimethylglycine dehydrogenase; DNMT: DNA-methyltransferase; FTD: 10-formyltetrahydrofolate dehydrogenase; FTS: 10-formyltetrahydrofolate synthase; GCS: γ-glutamylcysteine synthetase; GDC: glycine decarboxylase (glycine cleavage system); GNMT: glycine N-methyltransferase; GPX: glutathione peroxidase; GR: glutathione reductase; GS: glutathione synthetase; MAT-I: methionine adenosyl transferase I; MAT-III: methionine adenosyl transferase III; MS: methionine synthase; MTCH: 5,10-methenyltetrahydrofolate cyclohydrolase; MTD: 5,10-methylenetetrahydrofolate dehydrogenase; MTHFR: 5,10-methylenetetrahydrofolate reductase; NE: non-enzymatic conversion; PGT: Phosphoribosyl glycinamidetransformalase; SAAH: S-adenosylhomocysteine hydrolase; SDH: sarcosine dehydrogenase; SHMT: serinehydroxymethyltransferase; TS: thymidylate synthase; V_X: _velocity of the reaction catalyzed by X; **Metabolites**. 10f-THF: 10-formyltetrahydrofolate; 5mTHF: 5-methyltetrahydrofolate; AICAR: P-ribosyl-5-amino-4-imidazole carboxamide; CH = THF: 5-10-methenyltetrahydrofolate; CH2-THF: 5-10-methylenetetrahydrofolate; Cys: cysteine Cysta cystathionine; DHF: dihydrofolate; DMG: dimethylglycine; dTMP: deoxythymidine monophosphate; dUMP: deoxyuridine monophophate; GAR: glycinamide ribonucleotide; Glut: glutamate; Glut:Cys glutamyl-cysteine; Gly: glycine. GSH: glutathione; GSSG: glutathione disulfide; H_2_C= O: formaldehyde. H_2_O_2 _hydrogen peroxide. HCOOH formate. Hcy homocysteine. Met methionine. NADPH nicotinamide adenine dinucleotide phosphate; SAH: S-adenosylhomocysteine; SAM: S-adenosylmethionine; Sarc: sarcosine; Ser: serine; THF: tetrahydrofolate

## Competing interests

The authors declare that they have no competing interests.

## Authors' contributions

HN, MR, JJ, and CM formulated the questions and contributed substantially to the design of the project. RT and JP wrote the code and conducted the *in silico *experiments with HN and MR. The initial manuscript was written by RT, JP, MR, and HN. All authors read the manuscript, contributed revisions, and approved the submitted manuscript.

## Supplementary Material

Additional file 1 Supplementary Material – Model Details for A Mathematical Model of Glutathione Metabolism. A full description of the mathematical model is given. Click here for file
