# Supplementary material for: A mathematical model of glutathione metabolism
Source: Theor Biol Med Model. 2008 Apr 28;5:8. doi: 10.1186/1742-4682-5-8 (PMC2391141; doi:10.1186/1742-4682-5-8)
Supplement: Additional file 1 — Supplementary Material – Model Details for A Mathematical Model of Glutathione Metabolism. A full description of the mathematical model is given. [file 1742-4682-5-8-S1.pdf]

Supplementary Material – Model Details

for

A Mathematical Model of Glutathione Metabolism

Michael C. Reed[1]*, Rachel L. Thomas [1], Jovana Pavisic [1,2], S. Jill James[3], Cornelia M. Ulrich[4], H. Frederik Nijhout [2]

Address: [1] Department of Mathematics, Duke University, Durham, NC 27708, [2] Department of Biology, Duke University, Durham, NC 27708, [3] Department of Pediatrics, University of Arkansas for Medical Sciences, Little Rock, AK 72205, [4] Fred Hutchinson Cancer Research Center, Seattle, WA 98109-1024.

Email: Michael C. Reed - reed@math.duke.edu, Rachel L. Thomas - rachel@math.duke.edu, Jovana Pavisic - jovana.pavisic@duke.edu, S. Jill James - JamesJill@uams.edu, Cornelia M. Ulrich - nulrich@fhcrc.org, , H. Frederik Nijhout - hfn@duke.edu

* Corresponding author: Michael C. Reed

The model consists of 34 differential equations that express the rate of change of each of the substrates in the rectangular boxes in Figure 1. Each of the differential equations is a mass balance equation that says that the time rate of change of the particular metabolite is the sum of the rates at which it is being made minus the rates it is being consumed in biochemical reactions plus or minus net transport rates from other compartments. In order to display the differential equations in a coherent and understandable way, we have chosen notation for the variables and reaction rates that is both more uniform and more spare than some notation commonly in use, for example the concentration of 5-methyltetrahydrofolate is denoted 5mf instead of the usual [5mTHF]. For simplicity, we will refer to the transsulfuration pathway, the synthesis of glutathione, the transport of glutathione out of the cell and its breakdown in the blood as "glutathione metabolism." All of this notation is described in Part A, below. In Part B we give the differential equations, which are written in terms of reaction and transport rates. In Part C the kinetic formulas and constants for these reaction and transport rates are given with some justifications. Part D describes how the model works, what is given and what is computed, in particular experiments.

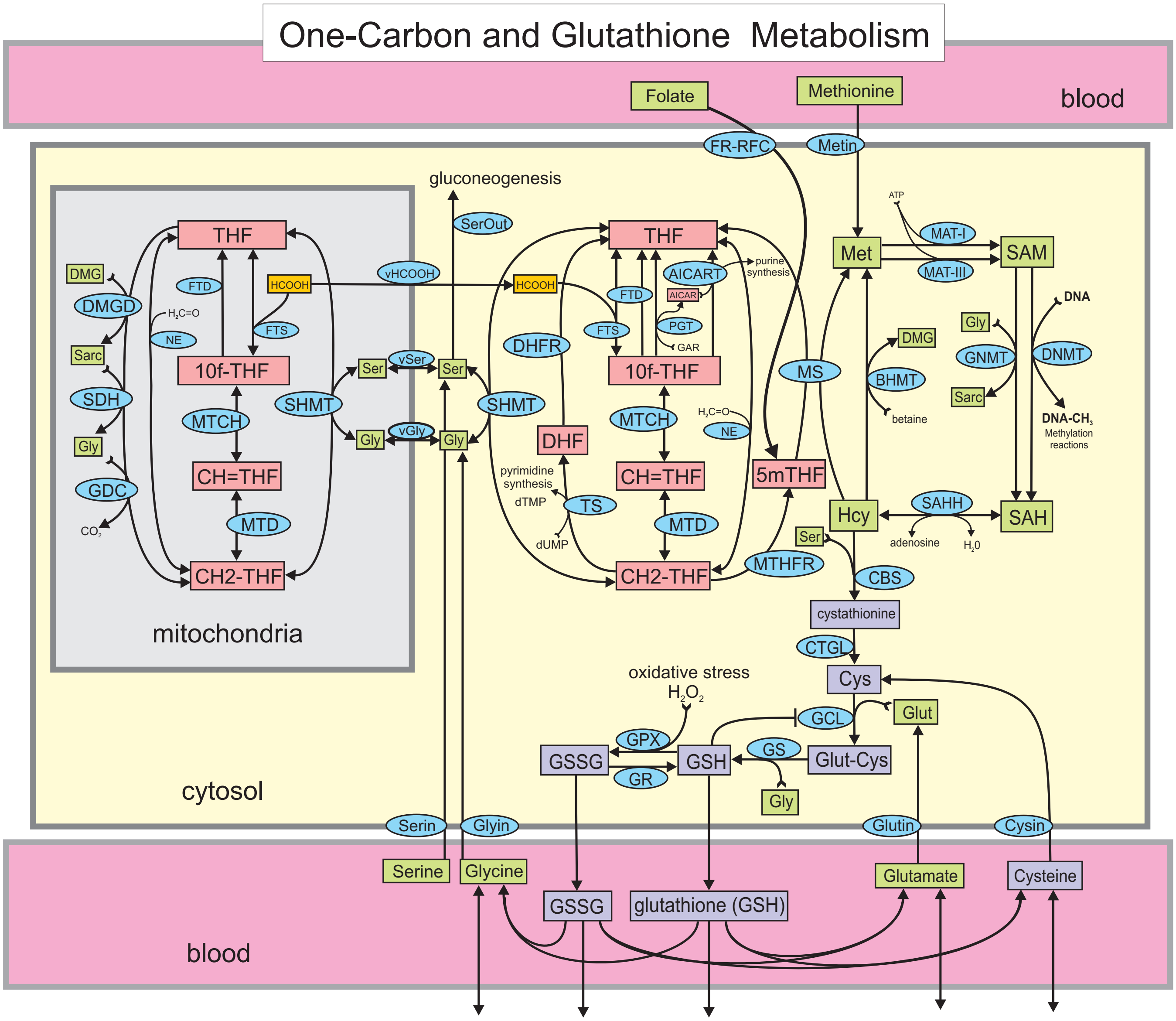

Figure 1. One-carbon and Glutathione Metabolism. Rectangular boxes indicate substrates that are variables in the model. Non-boxed substrates are either held constant in the model or are products of reactions and are not kept track of. Arrows at the bottom of the figure represent import from the gut and other cells, and losses to other cells and to degradation. Each internal arrow indicates a biochemical reaction or transport reaction. The ellipses contain the acronyms of enzymes or transporters.

Part A: Notation.

The complete names of the enzymes indicated by acronyms in Figure 1 are as follows.

Table S1: Enzyme names and acronyms.

mitochondrial folate cycle enzymes

$_\mathrm{m}$SHMT	serinehydroxymethyltransferase
$_\mathrm{m}$FTD	10-formyltetrahydrofolate dehydrogenase
$_\mathrm{m}$FTS	10-formyltetrahydrofolate synthase
$_\mathrm{m}$MTCH	5,10-methenyltetrahydrofolate cyclohydrolase
$_\mathrm{m}$MTD	5,10-methylenetetrahydrofolate dehydrogenase
$_\mathrm{m}$NE	non-enzymatic conversion
DMGD	dimethylglycine dehydrogenase
SDH	sarcosine dehydrogenase
GDC	glycine decarboxylase (glycine cleavage system)

cytosolic folate cycle enzymes

$_\mathrm{c}$SHMT	serinehydroxymethyltransferase
DHFR	dihydrofolate reductase
$_\mathrm{c}$FTD	10-formyltetrahydrofolate dehydrogenase
$_\mathrm{c}$FTS	10-formyltetrahydrofolate synthase
$_\mathrm{c}$MTCH	5,10-methenyltetrahydrofolate cyclohydrolase
$_\mathrm{c}$MTD	5,10-methylenetetrahydrofolate dehydrogenase
MTHFR	5,10-methylenetetrahydrofolate reductase
AICART	aminoimidazolecarboxamide ribonucleotide transferase
PGT	Phosphoribosyl glycinamidetransformalase
$_\mathrm{c}$NE	non-enzymatic conversion
TS	thymidylate synthase

methionine cycle enzymes

MAT-I	methionine adenosyl transferase I
MAT-III	methionine adenosyl transferase III
GNMT	glycine N-methyltransferase
DNMT	DNA-methyltransferase
SAAH	S-adenosylhomocysteine hydrolase
MS	methionine synthase
BHMT	betaine-homocysteine methyltransferase
CBS	cystathionine β-synthase

glutathione metabolism enzymes

CTGL	γ-cystathionase
GCS	γ-glutamylcysteine synthetase
GS	glutathione synthetase
GPX	glutathione peroxidase
GR	glutathione reductase

We will use lower case three letter abbreviations for the concentrations of metabolites (μM). A prefix of m, c, or b, for mitochondria, cytosol, or blood, indicates the compartment. Metabolites occuring in only one compartment (like met), or metabolites whose concentrations are asumed equal in different compartments (like dmg and src) have no prefixes.

Table S2: Names of variables (μM).

mitochondrial folate cycle metabolites

mthf	tetrahydrofolate
m2cf	5-10-methylenetetrahydrofolate
m1cf	5-10-methenyltetrahydrofolate
m10f	10-formyltetrahydrofolate

cytosolic folate cycle metabolites

cthf	tetrahydrofolate
c2cf	5-10-methylenetetrahydrofolate
c1cf	5-10-methenyltetrahydrofolate
c10f	10-formyltetrahydrofolate
dhf	dihydrofolate
5mf	5-methyltetrahydrofolate
aic	P-ribosyl-5-amino-4-imidazole carboxamide

methionine cycle metabolites

met	methionine
sam	S-adenosylmethionine
sah	S-adenosylhomocysteine
hcy	homocysteine

glutathione metabolites

cyt	cystathionine
ccys	cytosolic cysteine
bcys	blood cysteine
glc	glutamyl-cysteine
cgsh	cytosolic glutathione
bgsh	blood glutathione
cgsg	cytosolic glutathione disulfide
bgsg	blood glutathione disulfide

other metabolites

bgly	blood glycine
bglu	blood glutamate
mgly	mitochondrial glycine
mser	mitochondrial serine
mcoo	mitochondrial formate

cglu	cytosolic glutamate
cgly	cytosolic glycine
cser	cytosolic serine
cCOO	cytosolic formate
dmg	dimethylglycine
src	sarcosine

Table S3: Names of constants (μM).

GAR	10	glycinamide ribonucleotide
NADPH	50	nicotinamide adenine dinucleotide phosphate
BET	50	betaine
HCHO	500	formaldehyde
DUMP	20	deoxyuridine monophophate
bser	150	blood serine (varies in some experiments)
bmet	30	blood methionine (varies in some experiments)
FOL	20	total cellular folate (varies in some experiments)
ssH2O2	.01	normal cellular hydrogen peroxide
H2O2	.01	cellular hydrogen peroxide (varies in some experiments)

It is assumed that total cellular folate is equally divided between the mitochondria and the cytosol [11] and that the mitochondria occupy one quarter of the volume of the cell. Thus the total normal folate concentration in the mitochondria is 40 μM, and in the cytosol is 13.3 μM. In the experiments in this paper folate is neither transported into the cell nor catabolized.

Part B: The Equations.

For each of the biochemical reactions indicated by a reaction arrow in Figure 1, we denote the velocity of the reaction (in μM/hr) by a capital V whose subscript is the acronym for the enzyme that catalyzes the reaction. Thus, for example, the velocity of the methionine synthase reaction is denoted by V_{MS}. Each of these velocities depends on the current values one or more of the variables (metabolite concentrations) and possibly also on one or more of the constants. Velocities of reactions that occur in both the mitochondria and cytosol will be distinguished by c and m, for example, $V_{\mathrm{c}SHMT}$ and $V_{\mathrm{m}SHMT}$.

$$
\begin{aligned}
\left(\frac{d}{dt}\right)\mathrm{mthf} &= V_{\mathrm{mFTD}}(\mathrm{m10f}) - V_{\mathrm{mSHMT}}(\mathrm{mser}, \mathrm{mthf}, \mathrm{mgly}, \mathrm{m2cf}) - V_{\mathrm{mFTS}}(\mathrm{mthf}, \mathrm{mcoo}, \mathrm{m10f}) \\
&\quad -V_{\mathrm{mNE}}(\mathrm{mthf}, \mathrm{HCHO}, \mathrm{m2cf}) - V_{\mathrm{GDC}}(\mathrm{mgly}, \mathrm{mthf}) - V_{\mathrm{SDH}}(\mathrm{msrc}, \mathrm{mthf}) \\
&\quad -V_{\mathrm{DMGD}}(\mathrm{mdmg}, \mathrm{mthf}) \\
\left(\frac{d}{dt}\right)\mathrm{m2cf} &= V_{\mathrm{mSHMT}}(\mathrm{mser}, \mathrm{mthf}, \mathrm{mgly}, \mathrm{m2cf}) + V_{\mathrm{mNE}}(\mathrm{mthf}, \mathrm{HCHO}, \mathrm{m2cf}) + V_{\mathrm{GDC}}(\mathrm{mgly}, \mathrm{mthf}) \\
&\quad -V_{\mathrm{mMTD}}(\mathrm{m2cf}, \mathrm{m1cf}) + V_{\mathrm{SDH}}(\mathrm{msrc}, \mathrm{mthf}) + V_{\mathrm{DMGD}}(\mathrm{mdmg}, \mathrm{mthf}) \\
\left(\frac{d}{dt}\right)\mathrm{m1cf} &= V_{\mathrm{mMTD}}(\mathrm{m2cf}, \mathrm{m1cf}) - V_{\mathrm{mMTCH}}(\mathrm{m1cf}, \mathrm{m10f}) \\
\left(\frac{d}{dt}\right)\mathrm{m10f} &= V_{\mathrm{mFTS}}(\mathrm{mthf}, \mathrm{mcoo}, \mathrm{m10f}) + V_{\mathrm{mMTCH}}(\mathrm{m1cf}, \mathrm{m10f}) - V_{\mathrm{mFTD}}(\mathrm{m10f}) \\
\left(\frac{d}{dt}\right)\mathrm{cthf} &= V_{\mathrm{MS}}(\mathrm{hcy}, \mathrm{5mf}, \mathrm{H2O2}, \mathrm{ssH2O2}) + V_{\mathrm{DHFR}}(\mathrm{dhf}, \mathrm{NADPH}) + V_{\mathrm{cFTD}}(\mathrm{c10f}) + V_{\mathrm{PGT}}(\mathrm{c10f}, \mathrm{GARP}) \\
&\quad -V_{\mathrm{cFTS}}(\mathrm{cthf}, \mathrm{ccoo}, \mathrm{c10f}) - V_{\mathrm{cSHMT}}(\mathrm{cser}, \mathrm{cthf}, \mathrm{cgly}, \mathrm{c2cf}) - V_{\mathrm{cNE}}(\mathrm{mthf}, \mathrm{HCHO}, \mathrm{m2cf}) \\
\left(\frac{d}{dt}\right)\mathrm{c2cf} &= V_{\mathrm{cSHMT}}(\mathrm{cser}, \mathrm{cthf}, \mathrm{cgly}, \mathrm{c2cf}) + V_{\mathrm{cNE}}(\mathrm{cthf}, \mathrm{HCHO}, \mathrm{c2cf}) - V_{\mathrm{TS}}(\mathrm{DUMP}, \mathrm{c2cf}) \\
&\quad -V_{\mathrm{MTHFR}}(\mathrm{c2cf}, \mathrm{NADPH}, \mathrm{sam}, \mathrm{sah}) - V_{\mathrm{cMTD}}(\mathrm{c2cf}, \mathrm{c1cf}) \\
\left(\frac{d}{dt}\right)\mathrm{c1cf} &= V_{\mathrm{cMTD}}(\mathrm{c2cf}, \mathrm{c1cf}) - V_{\mathrm{cMTCH}}(\mathrm{c1cf}, \mathrm{c10f}) \\
\left(\frac{d}{dt}\right)\mathrm{c10f} &= V_{\mathrm{cFTS}}(\mathrm{cthf}, \mathrm{ccoo}, \mathrm{c10f}) + V_{\mathrm{cMTCH}}(\mathrm{c1cf}, \mathrm{c10f}) - V_{\mathrm{cFTD}}(\mathrm{c10f}) \\
&\quad -V_{\mathrm{ART}}(\mathrm{c10f}, \mathrm{aic}) - V_{\mathrm{PGT}}(\mathrm{c10f}, \mathrm{GARP}) \\
\left(\frac{d}{dt}\right)\mathrm{dhf} &= V_{\mathrm{TS}}(\mathrm{DUMP}, \mathrm{c2cf}) - V_{\mathrm{DHFR}}(\mathrm{dhf}, \mathrm{NADPH}) \\
\left(\frac{d}{dt}\right)\mathrm{5mf} &= V_{MTHFR}(\mathrm{c2cf}, \mathrm{NADPH}, \mathrm{sam}, \mathrm{sah}) - V_{\mathrm{MS}}(\mathrm{hcy}, \mathrm{5mf}, \mathrm{H2O2}, \mathrm{ssH2O2}) \\
\left(\frac{d}{dt}\right)\mathrm{aic} &= V_{\mathrm{PGT}}(\mathrm{c10f}, \mathrm{GARP}) - V_{\mathrm{ART}}(\mathrm{c10f}, \mathrm{aic})
\end{aligned}
$$

$$\left(\frac{d}{dt}\right)\text{met} = V_{\text{BHMT}}(\text{hcy}, \textsc{bet}, \text{sam}, \text{sah}, \textsc{h2o2}, \text{ssH2O2}) + V_{\text{MS}}(\text{hcy}, \text{5mf}, \textsc{h2o2}, \text{ssH2O2}) + V_{\text{bMetc}}(\text{bmet}, \text{met}) - V_{\text{MATI}}(\text{met}, \text{sam}, \text{gsg}) - V_{\text{MATIII}}(\text{met}, \text{sam}, \text{gsg})$$

$$\left(\frac{d}{dt}\right)\text{sam} = V_{\text{MATI}}(\text{met}, \text{sam}, \text{gsg}) + V_{\text{MATIII}}(\text{met}, \text{sam}, \text{gsg}) - V_{\text{GNMT}}(\text{sam}, \text{sah}, \text{5mf}, \text{cgly}) - V_{\text{DNMT}}(\text{sam}, \text{sah})$$

$$\left(\frac{d}{dt}\right)\text{sah} = V_{\text{GNMT}}(\text{sam}, \text{sah}, \text{5mf}, \text{cgly}) + V_{\text{DNMT}}(\text{sam}, \text{sah}) - V_{\text{SAHH}}(\text{sah}, \text{hcy})$$

$$\left(\frac{d}{dt}\right)\text{hcy} = V_{\text{SAHH}}(\text{sah}, \text{hcy}) - V_{\text{CBS}}(\text{hcy}, \text{sam}, \text{sah}, \text{cser}, \textsc{h2o2}, \text{ssH2O2}) - V_{\text{BHMT}}(\text{hcy}, \textsc{bet}, \text{sam}, \text{sah}, \textsc{h2o2}, \text{ssH2O2}) - V_{\text{MS}}(\text{hcy}, \text{5mf}, \textsc{h2o2}, \text{ssH2O2})$$

$$\left(\frac{d}{dt}\right)\text{mgly} = V_{\text{mSHMT}}(\text{mser}, \text{mthf}, \text{mgly}, \text{m2cf}) - 3V_{\text{mGLYc}}(\text{cgly}, \text{mgly}) - V_{\text{GDC}}(\text{mgly}, \text{mthf}) + V_{\text{SDH}}(\text{msrc}, \text{mthf})$$

$$\left(\frac{d}{dt}\right)\text{mser} = -3V_{\text{mSERc}}(\text{cser}, \text{mser}) - V_{\text{mSHMT}}(\text{mser}, \text{mthf}, \text{mgly}, \text{m2cf})$$

$$\left(\frac{d}{dt}\right)\text{mcoo} = -3V_{\text{mHCOOHc}}(\text{ccoo}, \text{mcoo}) - V_{\text{mFTS}}(\text{mthf}, \text{mcoo}, \text{m10f})$$

$$\left(\frac{d}{dt}\right)\text{cgly} = V_{\text{bGLYc}}(\text{cgly}, \text{bgly}) + V_{\text{cSHMT}}(\text{cser}, \text{cthf}, \text{cgly}, \text{c2cf}) + V_{\text{mGLYc}}(\text{cgly}, \text{mgly}) - V_{\text{GNMT}}(\text{sam}, \text{sah}, \text{5mf}, \text{cgly}) - V_{\text{GS}}(\text{cgly}, \text{glc})$$

$$\left(\frac{d}{dt}\right)\text{cser} = V_{\text{bSERc}}(\text{bser}, \text{cser}) + V_{\text{mSERc}}(\text{cser}, \text{mser}) - V_{\text{cSHMT}}(\text{cser}, \text{cthf}, \text{cgly}, \text{c2cf}) - V_{\text{CBS}}(\text{hcy}, \text{sam}, \text{sah}, \text{cser}, \textsc{h2o2}, \text{ssH2O2}) - (1.2)\text{cser}$$

$$\left(\frac{d}{dt}\right)\text{ccoo} = V_{\text{mHCOOHc}}(\text{ccoo}, \text{mcoo}) - V_{\text{cFTS}}(\text{cthf}, \text{ccoo}, \text{c10f})$$

$$\left(\frac{d}{dt}\right)\text{dmg} = V_{\text{BHMT}}(\text{hcy}, \textsc{bet}, \text{sam}, \text{sah}, \textsc{h2o2}, \text{ssH2O2}) - V_{\text{DMGD}}(\text{mdmg}, \text{mthf})$$

$$\left(\frac{d}{dt}\right)\text{src} = V_{\text{GNMT}}(\text{sam}, \text{sah}, \text{5mf}, \text{cgly}) + V_{\text{DMGD}}(\text{mdmg}, \text{mthf}) - V_{\text{SDH}}(\text{msrc}, \text{mthf})$$

$$\left(\frac{d}{dt}\right)\mathrm{cyt} = V_{\mathrm{CBS}}(\mathrm{hcy}, \mathrm{sam}, \mathrm{sah}, \mathrm{cser}, \mathrm{H2O2}, \mathrm{ssH2O2}) - V_{\mathrm{CTGL}}(\mathrm{cyt})$$

$$\left(\frac{d}{dt}\right)\mathrm{ccys} = V_{\mathrm{CTGL}}(\mathrm{cyt}) - V_{\mathrm{GCS}}(\mathrm{ccys}, \mathrm{cglu}, \mathrm{cgsh}, \mathrm{glc}, \mathrm{H2O2}, \mathrm{ssH2O2}) + V_{\mathrm{bCYSc}}(\mathrm{ccys}, \mathrm{bcys}) - (.35)\frac{[\mathrm{ccys}]^2}{200}$$

$$\left(\frac{d}{dt}\right)\mathrm{cglu} = V_{\mathrm{bGLUc}}(\mathrm{cglu}, \mathrm{bglu}) - V_{\mathrm{GCS}}(\mathrm{ccys}, \mathrm{cglu}, \mathrm{cgsh}, \mathrm{glc}, \mathrm{H2O2}, \mathrm{ssH2O2}) - (.07)\mathrm{glu}$$

$$\left(\frac{d}{dt}\right)\mathrm{glc} = V_{\mathrm{GCS}}(\mathrm{ccys}, \mathrm{cglu}, \mathrm{cgsh}, \mathrm{glc}, \mathrm{H2O2}, \mathrm{ssH2O2}) - V_{\mathrm{GS}}(\mathrm{cgly}, \mathrm{glc}, \mathrm{gsh})$$

$$\begin{aligned}\left(\frac{d}{dt}\right)\mathrm{cgsh} &= V_{\mathrm{GS}}(\mathrm{cgly}, \mathrm{glc}, \mathrm{gsh}) - V_{\mathrm{cgshHb}}(\mathrm{cgsh}) - V_{\mathrm{cgshLb}}(\mathrm{cgsh}) \\ &\quad -(2)V_{\mathrm{GPX}}(\mathrm{cgsh}, \mathrm{H2O2}) + (2)V_{\mathrm{GR}}(\mathrm{cgsg}, \mathrm{NADPH}) - (.002)\mathrm{cgsh}\end{aligned}$$

$$\begin{aligned}\left(\frac{d}{dt}\right)\mathrm{cgsg} &= V_{\mathrm{GPX}}(\mathrm{cgsh}, \mathrm{H2O2}) - V_{\mathrm{GR}}(\mathrm{cgsg}, \mathrm{NADPH}) \\ &\quad -V_{\mathrm{cgsgHb}}(\mathrm{cgsg}) - V_{\mathrm{cgsgLb}}(\mathrm{cgsg}) - (.1)\mathrm{cgsg}\end{aligned}$$

$$\left(\frac{d}{dt}\right)\mathrm{bgly} = (90)\mathrm{bgsh} + (2)(67.5)\mathrm{bgsg} + V_{\mathrm{oGLYb}} - V_{\mathrm{bGLYc}}(\mathrm{cgly}, \mathrm{bgly}) - (.1)\mathrm{bgly}$$

$$\left(\frac{d}{dt}\right)\mathrm{bcys} = (90)\mathrm{bgsh} + (2)(67.5)\mathrm{bgsg} + V_{\mathrm{oCYSb}} - V_{\mathrm{bCYSc}}(\mathrm{ccys}, \mathrm{bcys}) - (.35)\mathrm{bcys}$$

$$\left(\frac{d}{dt}\right)\mathrm{bgsh} = V_{\mathrm{cgshHb}}(\mathrm{cgsh}) + V_{\mathrm{cgshLb}}(\mathrm{cgsh}) - (.7)\mathrm{bgsh} - (90)\mathrm{bgsh}$$

$$\left(\frac{d}{dt}\right)\mathrm{bgsg} = V_{\mathrm{cgsgHb}}(\mathrm{cgsg}) + V_{\mathrm{cgsgLb}}(\mathrm{cgsg}) - (7.5)\mathrm{bgsg} - (67.5)\mathrm{bgsg}$$

$$\left(\frac{d}{dt}\right)\mathrm{bglu} = (90)\mathrm{bgsh} + (2)(67.5)\mathrm{bgsg} + V_{\mathrm{oGLUb}} - V_{\mathrm{bGLUc}}(\mathrm{cglu}, \mathrm{bglu}) - (.1)\mathrm{bglu}$$

Part C: Kinetics.

C.1 Reactions with standard kinetics. For many of the velocities, we assume that their dependence on substrates has Michaelis-Menten form with one substrate

$$V = \frac{V_{max}[S]}{K_m + [S]},$$

or random order Michaelis-Menten form with two substrates:

$$V = \frac{V_{max}[S_1][S_2]}{(K_{m,1} + [S_1])(K_{m,2} + [S_2])}.$$

Some reactions, for example V_{cSHMT}, are assumed to have reversible random order Michaelis-Menten kinetics with two substrates in each term. For all these velocities, the form is clear and the K_m and V_{max} values appear in Table S4, below, along with references. Enzymes that occur in both the mitochondria and the cytosol are assumed to have the same form of the kinetics in both compartments. If the kinetic constants differ in the cytosol and the mitochondria that is indicated by the prefixes m and c, respectively.

Table S4. Model kinetic parameter values (time in hrs., concentrations in μM).

Parameter	Literature	Model	Reference
**AICART**			
$K_{\mathrm{m,10f}}$	5.9-50	5.9	[80][72][76][61]
$K_{\mathrm{m,aic}}$	10-100	100	[80][72][61]
V_{max}		55000	
**CTGL.**			
$K_{m,\mathrm{cyt}}$	500	500	[74]
V_{max}	-	1500	
**DHFR.**			
$K_{\mathrm{m,dhf}}$	0.12-1.9	0.5	[36][80][72][5]
$K_{\mathrm{m,NADPH}}$	0.3-5.6	4.0	[36][80][72][5]
V_{max}	350-23000	2000	[36][80][72]
**DMGD**			
$K_{\mathrm{m,dmg}}$	50	50	[60]
$K_{\mathrm{m,mthf}}$	-	50	
V_{max}	-	15000	

Parameter	Literature	Model	Reference
**FTD.**			
$K_{\mathrm{m,10f}}$	0.9-20	20	[67][40]
$_{\mathrm{c}}V_{max}$		500	
$_{\mathrm{m}}V_{max}$		1050	
**$_{\mathrm{c}}$FTS.**			
$K_{\mathrm{m,thf}}$	0.1 - 600	3	[80][76]
$K_{\mathrm{m,coo}}$	8 - 1000	43	[80][76]
V_{max}	100 - 486000	3900	[80][76]
**$_{\mathrm{m}}$FTS.**			
$K_{\mathrm{m,thf}}$	0.1 - 600	3	[80][76]
$K_{\mathrm{m,coo}}$	8 - 1000	43	[80][76]
V_{max}	100 - 486000	2000	[80][76]
$K_{\mathrm{m,10f}}$	-	22	
V_{max}	-	6300	
**GDC**			
$K_{\mathrm{m,_mgly}}$	3400-40000	3400	[79][31][29][22]
$K_{\mathrm{m,_mthf}}$	50	50	[24]
V_{max}	-	15000	
**GPX.**			
$K_{m,\mathrm{gsh}}$	1330	1330	[9]
$K_{m,\mathrm{H2O2}}$	-	.09	
V_{max}	-	4500	
**GR.**			
$K_{m,\mathrm{gsg}}$	72, 107	107	[44], [65]
$K_{m,\mathrm{NADPH}}$	10.4	10.4	[44]
V_{max}	-	8925	
**MTCH.** (positive direction is from 1cf to 10f)			
$K_{\mathrm{m,1cf}}$	4-250	250	[80][72][76]
cV_{max}	880-1380000	500000	[72][76]
mV_{max}	880-1380000	790000	[72][76]
$K_{\mathrm{m,10f}}$	20-450	100	[80][72][76]
V_{max}	10.5-1380000	20000	[72][76]

Parameter	Literature	Model	Reference
**MTD.** (positive direction is from 2cf to 1cf)			
$K_{\mathrm{m,2cf}}$	2-5	2	[80][76]
cV_{max}	520-594000	80000	[36][80][76]
mV_{max}	520-594000	180000	[36][80][76]
$K_{\mathrm{m,1cf}}$	1-10	10	[82][76]
V_{max}	594000	600000	[76]
**PGT.**			
$K_{m,\mathrm{10f}}$	4.9-58	4.9	[80][72][7][8]
$K_{\mathrm{m,GAR}}$	520	520	[80][72][7][8]
V_{max}	6600-16200	24300	[80][72][7][8]
**SAHH.** (positive direction is from sah to hcy)			
$K_{\mathrm{m,sah}}$	0.75-15.2	6.5	[15][41][23][30]
V_{max}	-	320	
$K_{\mathrm{m,hcy}}$	56.6-200	150	[23] [26][30]
V_{max}	-	4530	
**SDH**			
$K_{\mathrm{m,src}}$	320	320	[21]
$K_{\mathrm{m,mthf}}$	-	50	
V_{max}	-	15000	
**SHMT.** (positive direction is from thf to 2cf)			
$K_{\mathrm{m,ser}}$	350-1300	600	[80][72][76][13][66]
$K_{\mathrm{m,thf}}$	45-300	50	[80][72][76][68][69]
cV_{max}	500-162000	5200	[36][72][76][69]
mV_{max}	500-162000	11440	[36][72][76][69]
$K_{\mathrm{m,gly}}$	3000-10000	10000	[36][80][72][76][66]
$K_{\mathrm{m,2cf}}$	3200-10000	3200	[36][72][76][68]
cV_{max}	12600-120000000	15000000	[36][72][76]
mV_{max}	12600-120000000	30000000	[36][72][76]
**TS.**			
$K_{\mathrm{m,DUMP}}$	5-37	6.3	[36][80][10][45]
$K_{\mathrm{m,2cf}}$	10-45	14	[36][80][10][45]
V_{max}	30-4200	5000	[72][45]

C.2 Reactions with nonstandard kinetics. We now discuss the reactions where the kinetics have a special form.

BHMT. The kinetics of BHMT are Michaelis-Menten with the parameters $K_{m,1} = 12$, $K_{m,2} = 100$, and $V_{max} = 2160$ [16],[71]. The form of the inhibition of BHMT by SAM was

derived by non-linear regression on the data of [17] and scaled so that it equals 1 when the external methionine concentration is 30 μM. The last factor represents the inhibition of BHMT by oxidative stress, see the discussion below. $\text{ssH2O2} = .01\mu$M is the steady-state concentration of H_2O_2 and and H2O2 is the current concentration (set differently in different experiments). $K_i = .01\mu$M is the inhibition constant. When $\text{H2O2} = \text{ssH2O2}$ the factor equals 1.

$$V_{\text{BHMT}} = e^{-.0021(\text{sam}+\text{sah})} e^{+.0021(102.6)} \frac{V_{max}(\text{hcy})(\text{BET})}{(K_{m,1} + \text{hcy})(K_{m,2} + \text{BET})} \left(\frac{\text{ssH2O2} + K_i}{\text{H2O2} + K_i} \right)$$

CBS. The kinetics of CBS are standard Michaelis-Menten with $K_{m,1} = 1000$ for hcy taken from [19] and $K_{m,2} = 2000$ for cser taken from [78], with $V_{max} = 700,000$. The form of the activation of CBS by sam and sah was derived by non-linear regression on the data in [37] and [42] and scaled so that it equals 1 when the external methionine concentration is 30 μM. The last factor represents the activation of CBS by oxidative stress, see the discussion below. $\text{ssH2O2} = .01\mu$M is the steady-state concentration of H_2O_2 and and H2O2 is the current concentration (set differently in different experiments). $K_a = .035\mu$M is the activation constant. If $\text{H2O2} = \text{ssH2O2}$ the factor equals 1.

$$V_{\text{CBS}} = \left(\frac{V_{max}(\text{hcy})(\text{cser})}{(K_{m,1} + \text{hcy})(K_{m,2} + \text{cser})} \right) \left(\frac{(1.086)(\text{sam} + \text{sah})^2}{(30)^2 + (\text{sam} + \text{sah})^2} \right) \left(\frac{\text{H2O2} + K_a}{\text{ssH2O2} + K_a} \right).$$

DNMT. The DNA methylation reaction is given as a uni-reactant scheme with sam as substrate. That is, the substrates for methylation are assumed constant. Their variation can be modeled by varying the V_{max}. The kinetic constants, $V_{max} = 180$, $K_m = 1.4$, and $K_i = 1.4$ are from [20].

$$V_{\text{DNMT}} = \frac{V_{max}(\text{sam})}{K_m(1 + \frac{\text{sah}}{K_i}) + \text{sam}}.$$

GCS. The reaction is reversible and GSH is a competitive inhibitor of GCS against glutamate [63],[70],[48]. The form of the kinetics is taken from [48] and the kinetic constants from [48], [32], [28]: $K_{\text{m,cys}} = 100$; $K_{\text{m,glut}} = 1900$; the equilibrium constant for the breakdown of the enzyme-glutamyl-cysteine complex, $K_e = 5597$; inhibition by GSH, $K_i = 8200$; the dissociation constant of glutamyl-cysteine, $K_p = 300$; and $V_{max} = 3600$. The last factor represents the activation of GCS by oxidative stress. $\text{ssH2O2} = .01\mu$M is the steady-state concentration of H_2O_2 and and H2O2 is the current concentration (set differently in different experiments). $K_a = .01\mu$M is the activation constant. When $\text{H2O2} = \text{ssH2O2}$ the factor = 1.

$$V_{\rm GCS} = \frac{V_{max}((\text{cys})(\text{glu}) - \frac{(\text{glc})}{K_e})}{K_{\rm m}^{\rm cys} K_{\rm m}^{\rm glu} + K_{\rm m}^{\rm cys}(\text{glu}) + K_{\rm m}^{\rm glu}(\text{cys})(1 + \frac{(\text{gsh})}{K_i} + \frac{(\text{glu})}{K_{\rm m}^{\rm glu}}) + \frac{(\text{glc})}{K_p} + \frac{(\text{gsh})}{K_i}} \left(\frac{\text{ssH2O2} + K_a}{\text{H2O2} + K_a}\right)$$

GNMT. The first factor of the GNMT reaction is standard Michaelis-Menten with $V_{max} = 245$, and $K_{m,\text{sam}} = 32$ and $K_{m,\text{gly}} = 130$ for cgly estimated from [53]. The second term is product inhibition by sah from [62] with $K_i = 18$. The third term, the long-range inhibition of GNMT by 5mf, was derived by non-linear regression on the data of [84], Figure 3.

$$V_{\rm GNMT} = \left(\frac{V_{max}(\text{sam})(\text{cgly})}{(K_{m,1} + \text{sam})(K_{m,2} + \text{cgly})}\right)\left(\frac{1}{1 + \frac{\text{sah}}{K_i}}\right)\left(\frac{4.8}{0.35 + 5\text{mf}}\right)$$

GS. We follow [48] and [32] and use a reversible bi-reactant Michaelis-Menten mechanism,

$$V_{\rm GS} = \frac{V_{max}((gly)(glc) - \frac{(gsh)}{K_e})}{K_m^{\rm glc} K_m^{\rm gly} + (glc)K_m^{\rm gly} + (gly)K_m^{\rm glc}(1 + \frac{(glc)}{K_m^{\rm glc}}) + \frac{(\text{gsh})}{K_p}}$$

with kinetic constants $K_m^{\rm glc} = 22$; $K_m^{\rm gly} = 300$, the dissociation constant for GSH, $K_p = 30$, the equilibrium constant for the overall reaction, $K_e = 5600$, and $V_{max} = 5400$.

MAT-I. The MAT-I kinetics are from [77], Table 1, and we take $V_{max} = 260$ and $K_m = 41$. The inhibition by sam was derived by non-linear regression on the data from [77], Figure 5. The last factor represents the inhibition of MATI by glutathione disulfide. The inhibition constant $K_i = 2140$ is taken from [58].

$$V_{\rm MAT\text{-}I} = \left(\frac{V_{max}(\text{met})}{K_m + \text{met}}\right)(0.23 + (0.8)e^{-(0.0026)(\text{sam})})\left(\frac{K_i + 66.71}{K_i + \text{gsg}}\right)$$

MAT-III. The methionine dependence of the MAT-III kinetics is from [64], Figure 5, fitted to a Hill equation with $V_{max} = 220$, $K_m = 300$. The activation by sam is from [77], Figure 5, fitted to a Hill equation with $K_a = 360$. The last factor represents the inhibition of MATI by glutathione disulfide with constant $K_i = 4030$ is taken from [58].

$$V_{\rm MAT\text{-}III} = \left(\frac{V_{max}(\text{met})^{1.21}}{K_m + (\text{met})^{1.21}}\right)\left(1 + \frac{(7.2)(\text{sam})^2}{(K_a)^2 + (\text{sam})^2}\right)\left(\frac{K_i + 66.71}{K_i + \text{gsg}}\right)$$

MS. We assume that the MS reaction has standard random bi-bi kinetics with $K_{\text{m,5mf}} = 25$ ([18][2]), $K_{\text{m,hcy}} = 1$([3]) and $V_{max} = 500$([3]). The last factor represents the inhibition of MS by oxidative stress, see the discussion below. $\text{ssH2O2} = .01\mu$M is the steady-state concentration of H_2O_2 and and H2O2 is the current concentration (set differently in different experiments). $K_i = .01\mu$M is the inhibition constant. When $\text{H2O2} = \text{ssH2O2}$ the factor equals 1.

$$V_{\text{MS}} = \frac{V_{max}(\text{5mf})(\text{hcy})}{(K_{\text{m,5mf}} + \text{5mf})(K_{\text{m,hcy}} + \text{hcy})} \left(\frac{\text{ssH2O2} + K_i}{\text{H2O2} + K_i} \right)$$

MTHFR. The first factor in the formula for the MTHFR reaction velocity

$$V_{\text{MTHFR}} = \frac{V_{max}(\text{2cf})(\text{NADPH})}{(K_{m,1} + \text{2cf})(K_{m,2} + \text{NADPH})} \cdot \frac{72}{10 + \text{sam} - \text{sah}}$$

is standard Michaelis-Menten with $K_{m,1} = 50$, $K_{m,2} = 16$, and $V_{max} = 5300$ taken from [47][27][13].

The inhibition of MTHFR by sam, the second factor, was derived by non-linear regression on the data of [38][81] and has the form $10/(10 + \text{sam})$. In addition, sah competes with sam for binding to the regulatory domain of MTHFR. It neither activates nor inhibits the enzyme [81] but prevents inhibition by sam; thus, we take our inhibitory factor to be:

$$I = \frac{10}{10 + \text{sam} - \text{sah}}.$$

The factor 72 scales the inhibition so that it has value 1 when the external methionine concentration is 30 μM.

NE. The kinetics of the non-enzymatic reversible reaction between thf and 2cf are taken to be mass action,

$$V_{\text{NE}} = k_1(\text{thf})(\text{HCHO}) - k_2(\text{2cf}).$$

The rate constants are $k_1 = 0.03$, and $k_2 = 22$ in the cytosol and $k_2 = 20$ in the mitochondria.

C.3 Transport kinetics. We now discuss the kinetics of transport between the compartments. The general formula for the kinetics of transport between the blood and the cytosol is taken to be

$$V = \frac{V_{max}[bAA]}{K_m + [bAA]} - k_{\text{out}}[cAA],$$

where AA stands for an amino acid and the prefixes b and c refer to the blood and cytosolic compartments, respectively. Thus the kinetics are Michaelis-Menten coming into the cell and linear going out. We take the kinetics of transport of serine and glycine between the

cytosol and mitochondria to be Michaelis-Menten in both directions and the transport of formate between these two compartments to be linear in both directions. The transport of amino acids into cells is accomplished by a relatively small number of transport systems each of which handles several amino acids. Each transporter is specialized to handle amino acids with particular ionic characteristics [59][46]. The transporters are saturable and the K_m values have been measured in many systems [39][73][6]. Relatively little is about the kinetics by which amino acids leak out of cells so we take this process to be linear. This linear rate also includes the loss of cytosolic amino acids to other metabolic processes not in the model (see Figure 1), for example use in protein synthesis. The differential equation for $_{\text{c}}$ser includes a term, -(1.2)$_{\text{c}}$ser, that represents the use of serine in gluconeogenesis. In the model, the methionine and serine concentrations in the blood are fixed at 30 μM and 150 μM, respectively, except in the experiments in Section D of the Results. Cysteine, glycine, and glutamate are transported into the blood from outside at constant rates of $V_{\text{oCYSb}} = 70\mu\text{M/hr}$, $V_{\text{oGLYb}} = 630\mu\text{M/hr}$, and $V_{\text{oGLUTb}} = 273\mu\text{M/hr}$, respectively. These rates are varied in Section D of the Results.

The velocities of transport from blood to cytosol or mitochondria to cytosol are given by the transparent notation V_{bSERc} and V_{mSERc}, respectively. The units are in μM/hr increase *in the cytosol*. Since the cytosol is assumed to have three times the volume of the mitochondria, one μM increase in the cytosol due to transport from the mitochondria means a 3 μM decrease in the mitochondria. This is the reason for the 3's in the differential equations involving transport into and out of the mitochondria and in the transport kinetics below.

Table S5. Parameter values for amino acid transporters (hours, μM).

Parameter	Model value	Parameter	Model value
V_{bGLYc}		V_{cGLYm}	
K_m	150	$K_{m,\text{cgly}}$	5700
V_{max}	4600	V_{max}	10000
k_{out}	1	$K_{m,\text{mgly}}$	5700
		V_{max}	10000/3
V_{bSERc}		V_{cSERm}	
K_m	150	$K_{m,\text{cser}}$	5700
V_{max}	2700	V_{max}	10000
k_{out}	1	$K_{m,\text{mser}}$	5700
		V_{max}	10000/3
V_{bMETc}		V_{mHCOOHc}	
K_m	150	k_{in}	100/3
V_{max}	913	k_{out}	100
k_{out}	1		
V_{bCYSc}		V_{bGlutc}	
K_m	2100	K_m	300

V_{max}	14950	V_{max}	28000
k_{out}	1	k_{out}	1

Although GSH and GSSG are exported from the liver both canalicularly (to the bile) and sinusoidally (to the blood), sinusoidal surfaces account for approximately 90% of the total membrane and canalicular efflux is minimal, [1], so we chose to include only sinusoidal export in our model. High and low affinity transporters operate to export GSH and GSSG, with high affinity transport dominating at low intracellular concentrations, and low affinity transport dominating at high intracellular concentrations. According to [57], high affinity transport accounts for approximately 12-17% of total transport at normal hepatic GSH levels (5,000 - 7,000 μM), and we used this figure to determine V_{max} values that appropriately balanced the two types of export. The high and low affinity transporters for GSH are denoted by V_{cgshHb} and V_{cgshLb}, respectively, and similar notation is used for the GSSG transporters. Under normal conditions (those reported in Tables 1-4 of the main paper), $V_{\text{cgshHb}} = 146\mu\text{M/hr}$ and $V_{\text{cgshLb}} = 1005\mu\text{M/hr}$.

The kinetics of sinusoidal efflux of GSH has been well studied in the perfused rat liver. The major part of the flux is carried by the low affinity transporter, which has sigmoidal kinetics with a Vmax in the range 900-1400 μM/hr, a Km of approximately 3000 μM, and a Hill coefficient of approximately 3 ([55][56][57]). In our model, we use a Vmax of 1100 μM/hr, a Km of 3000 μM, and a Hill coefficient of 3. We use standard Michaelis-Menten kinetics for the high affinity GSH transporter and for the two GSSG transporters. Parameter values are taken from [1][34][43][54][55][56][57].

Table S6. Parameter values for GSH and GSSG transporters (μM/hr).

Parameter	Model value	Parameter	Model value
V_{cgshHb}		V_{cgshLb}	
K_m	150	K_m	3000
V_{max}	150	V_{max}	1100
		Hill	3
V_{cgsgHb}		V_{cgsgLb}	
K_m	1250	K_m	7100
V_{max}	40	V_{max}	4025

C.4 Oxidative stress. Intracellular hydrogen peroxide levels and the ratio of the redox couple, GSH and GSSG, are the main indicators of oxidative stress in our model. Both H_2O_2 and GSSG play a role in regulating enzyme velocities in other parts of the methionine cycle

and the transsulfuration pathway. Hydrogen peroxide inhibits MS and BHMT, and activates CBS and GCL [14][50]. Glutathione disulfide inhibits MAT I and MAT III [58][12].

To add inhibition to our enzyme reactions, we multiplied the reaction velocity by the term

$$\frac{k_i + [S]_{ss}}{k_i + [S]}$$

where k_i is the inhibition constant, $[S]$ is the substrate concentration, and $[S]_{ss}$ is the concentration at steady-state. Since the inhibitor concentration is in the denominator, the reaction velocity decreases as the concentration of the substrate increases. We chose this format so that the velocity of the reaction at steady-state would remain the same once we added the inhibition. This allows us to compare the system with and without inhibition. For the inhibitions of MS and BHMT, the substrate is H_2O_2, and for MAT I and MAT III, the substrate is GSSG. We used the inhibition constants, 2140 and 4030, found in [58] for GSSG's inhibition of MAT I and MAT III, respectively. We were unable to find kinetic data for the inhibitions by H_2O_2 and so we chose our inhibition constants to be the value of steady-state intracellular H_2O_2 concentration so that the effects of the inhibitions would be nearly linear. For examples, see the kinetics of MS, BHMT, MatI, and MATIII.

For enzyme activation by H_2O_2, we used a similar approach. We multiplied the reaction velocity by the term

$$\frac{k_a + [S]}{k_a + [S]_{ss}}$$

Note that here the activator concentration is in the numerator, and so as the concentration of the substrate increases, so does the velocity of the reaction. Again, the multiplier is one at steady-state, allowing for comparison of the system with and without inhibition. For examples, see the kinetics of CBS and GCS.

C.5. GSH and GSSG breakdown in the blood. The last five differential equations in Part B are for the five variables that we keep track of in the blood, bcys, bgly, bgsh, bgsg and bglu. Since bcys, bgly, and bglu are variables, we can not specify them (as we do the concentrations bmet and bser). Instead we specify the import of cysteine, glycine, and glutamate from outside the system (for example, the gut) into the blood. The normal values of these constants are $V_{\text{oCYSb}} = 40\mu\text{M/hr}$, $V_{\text{oGLYb}} = 630\mu\text{M/hr}$, and $V_{\text{oGLUb}} = 273\mu\text{M/hr}$. These inputs are changed in various experiments and they vary in time in the experiments described in Results D.

The purpose of our model is to study the properties of intracellular glutathione metabolism, in particular the effects of oxidative stress and trisomy 21. Of course intracellular glutathione metabolism is affected by the import of amino acids and the export and usage of GSH and GSSG. We therefore need include a blood compartment and to keep track of bcys, bgly, bgsh, bgsg and bglu. The blood compartment is complicated because the blood interacts in important ways with the kidney, the brain, and other tissues. However, these important interactions are beyond the scope of this initial investigation. Therefore, in this model, we

make simple assumptions about the kinetics of amino acids in the blood compartment: each hour, 10% of the cysteine, glycine, and glutathione, are lost (for example to other tissues) and an additional 25% of cysteine is lost representing conversion to cystine; some GSH and GSSG are also lost but most GSG and GSSG in the blood is decomposed into the constituent amino acids.

Part D: *In silico* experimentation. The inputs to the model that must be specified are the constants in Table S3, the concentrations of methionine and serine in the blood, plus the rates of input of cysteine, glycine, and glutathione to the blood . If one starts with initial values for the variables and solves the differential equations (when the velocities have the formulas given in Part C) all the concentration variables and velocities eventually approach steady state. If the constants and inputs have their "normal" values as defined above, these steady state concentrations and the velocities are the ones given in Tables 1-4 in Results A. Most of the *in silico* experiments reported in the paper were done by starting the system at this steady state, changing one or more parameters, and letting the system relax to a new steady state. For example, in Results B we discuss our simulations of three different experimental situations: we lower amino acid inputs to 1/3 of normal to simulate fasting; we block the enzyme GCS, we block the enzyme CTGL. In other experiments particular enzyme inhibitions were added or removed, or particular constants, for example the H_2O_2 concentration, were changed. In Results D all the amino acid inputs are varied as functions of time (simulating meals). By solving the differential equations one can study how this large complex system responds to such fluctuating inputs.

References

[1] Aw, T.Y., Ookhtens, M., Ren, C., & Kaplowitz, N. (1986). Kinetics of glutathione efflux from isolated rat hepatocytes. *Am. J. Physiol. Gastrointest. Liver Physiol.* **250**, G236-G243.

[2] Banerjee, R. , Frasca, V., Ballou, D., and, Matthews, R., 1990. Participation of Cob(I)alamin in the reaction catalyzed by methionine synthase from *Escherichia coli*: a steady state and rapid reaction kinetic analysis. *Biochem.*, **29**, 11101-11109.

[3] Banerjee, R. , Chen, Z., and Gulati, S. 1997. Methionine synthase from pig liver. *Meth. Enzymol.*, **281**, 189-197.

[4] Bannai, S., & Tateishi, N. (1986). Role of membrane transport in metabolism and function of glutathione in mammals. *J. Membrane Biol.* **89**, 1-8.

[5] Blake, R. L.. (1995). Eukaryotic dihydrofolate reductase. *Adv. Enzymol.* **60**, 23.

[6] Boerner, P., Saier, M. H. (1982) Growth regulation and amino acid transport in epithelial cells: influence of culture conditions and transformation on A,ASC, and I transport activities. *J. Cell Physiol.*, **113**, 240-246.

[7] Caperelli, C.A. (1985). Mammalian glycinamide ribonucleotide transformylase: purification and some properties. *Biochemistry*,**24**, 1316-1320.

[8] Caperelli, C.A. (1989). Mammalian glycinamide ribonucleotide transformylase. Kinetic mechanism and associated de novo purine biosynthetic activities. *J. Biol. Chem.*,**264**, 5053-5057.

[9] Carmagnol, F., Sinet, P.M., & Jerome, H. (1983). Selenium-dependent and non-selenium-dependent glutathione peroxidases in human tissue extracts. *Biochim. Biophys. Acta.* **759**, 49-57.

[10] Carreras, C.W. and Santi, D.V. (1995). The catalytic mechanism and structure of thymidylate synthase. *Ann. Rev. Biochem.*,**64**, 721-762.

[11] Cook,R. J. (2001) Folate metabolism in *Homocysteine in Health and Disease* (Carmel, R., and Jacobsen, D. W., eds), pp. 113-134, Cambridge Univ. Press, Cambridge

[12] Corrales, F.J., Ruiz, F., Mato & J. (1999). *In vivo* regulation by glutathione of methionine adenosyltransferase S-nitrosylation in rat liver. *J. Hepatology* **31**, 887-894.

[13] Daubner, S.C. and Matthews, R.G. (1982). Purification and properties of methylenetetrahydrofolate reductase from pig liver.*J. Biol. Chem.*,**257**, 140-145.

[14] Deplancke, B. & Gaskins, H.R. (2002). Redox control of the transsulfuration and glutathione biosynthesis pathways. *Current Opinion in Clinical Nutrition and Metabolic Care* **5**, 85-92.

[15] Doskeland SO, Ueland PM. (1982) Comparison of some physicochemical and kinetic properties of S-adenosylhomocysteine hydrolase from bovine liver, bovine adrenal cortex and mouse liver. *Biochim Biophys Acta*, **708**:185-193.

[16] Finkelstein, J. D., Harris, B. J., and Kyle, W. E. 1972. Methionine metabolism in mammals: kinetic study of betaine-homocysteine methyltransferase, *Arch. Biochem. Biophys.*, **153**, 320-324.

[17] Finkelstein, J. D., and Martin, J. J. (1984) *Biochem. Biophys. Res. Comm.* **118**, 14-19

[18] Finkelstein, J. D., and Martin, J. J., 1986. Methionine metabolism in mammals: adaptation to methionine excess, *J. Biol. Chem.* **261**, 1582-1587.

[19] Finkelstein, J. (2001) Regulation of homocysteine metabolism in *Homocysteine in Health and Disease* (Carmel, R., and Jacobsen, D. W., eds), pp. 92-99, Cambridge Univ. Press, Cambridge

[20] Flynn, J., and Reich, N. (1998) *Biochemistry* **37**, 15162-15169

[21] Frisell, W.R., and Mackenzie, C.G. (1970) Sarcosine dehydrogenase and dimethylglycine dehydrogenase (rat liver, monkey liver), *Methods Enzymol.*, **17A**, 976-981

[22] Fujiwara, K., and Motokawa, Y. (1983) Mechanism of the glycine cleavage reaction: steady state kinetic studies of the P-protein-catalyzed reaction. *J. Biol. Chem.*, **258**, 8156-8162.

[23] Fujioka M, Takata Y ((1981): S-Adenosylhomocysteine Hydrolase from rat liver: Purification and some properties. *J Biol Chem*, **256**:1631-1635.

[24] Fujiwara, K., Okamura-Ikeda, K., and Motokawa, Y. (1984) Mechanism of the glycine cleavage reaction: further characterization of the intermediate attached to the H-protein and of the reaction catalyzed by the T-protein. *J. Biol. Chem.*, **259**, 10664-10668.

[25] Fukagawa, N., Ajami, A., & Young, V. (1996). Plasma methionine and cysteine kinetics in response to an intravenous glutathione infusion in adult humans. *Am. J. Physiol.* **270** *(Endocrinol Metab. 33)*, E209-E214.

[26] Gomi T, Takata Y, Date T, Motoji F, Akasamit RR, Backlund P Jr, Cantoni G (1990): Site-directed mutagenesis of rat liver S-Adenosylhomocysteine. *J Biol Chem*, **265**:16101-16107.

[27] Green, J. M., MacKenzie, R. E., & R. G. Matthews (1988). Substrate flux through methylenetetrahydrofolate dehydrogenase: Predicted effects of the concentration of methylenetetrahydrofolate on its partitioning into pathways leading to nucleotide biosynthesis or methionine regeneration. *Biochemistry* **27**, 8014-8022.

[28] Griffith, O.W. (1999). Biologic and pharmacologic regulation of mammalian glutathione synthesis. *Free Radical Biol. & Med.* **27**, 922-935.

[29] Gueguen, V., Macherel, D., Neuberger, M., Saint Pierre, C., Jaquinod, M., Gans, P., Douce, R., and Bourguignon, J. (1999) Structural and functional characterization of H protein mutants of the glycine decarboxylase complex. *J. Biol. Chem.*, **274**, 26344-26352.

[30] Hershfield M, Aiyar VN, Premakumar R, Small WC (1985): S-Adenosylhomocysteine hydrolase from human placenta. *J Biochem*, **230**, 43-52.

[31] Hiraga, K., and Kikuchi, G. (1980) The mitochondrial glycine cleavage system: purification and properties of glycine decarboxylase from chicken liver mitochondria, *J. Biol. Chem.*, **255**, 11664-11670.

[32] Hell R, and Bergmann L. (1990) γ-Glutamylcysteine synthestase in higher plants: catalytic properties and subcellular localization. *Planta*, **180**:603-612.

[33] Ichikawa A, Sato S, Tomita K (1985): Purification and characterization of S-adenosylhomocysteine hydrolase from mouse mastocytoma P-815 cells. Evidence for cell cycle-specific fluctuation of the enzyme activity. *J Biochem*, **97**: 189-197.

[34] Inoue, M., Kinne, R., Tran, T., & Arias, I. (1984). Glutathione transport across hepatocyte plasma membranes. *Eur. J. Biochem.* **138**, 491-495.

[35] Jackson, R.C. 1973. Kinetic simulation of anticancer drug interactions, *Int. J. Bio-Medical Comp.*, **11**, 197-224.

[36] Jackson, R.C. and Harrap K.R. (1973). Studies with a mathematical model of folate metabolism. *Arch. Biochem. Biophys.*, **158**, 827-841.

[37] Janosik, M., Kery, V., Gaustadnes, M., Maclean, K. N., and Kraus, J. P. (2001) *Biochemistry* **40**, 10625-10633

[38] Jencks, D. A., and Matthews, R. G. (1987) *J. Biol. Chem.* **262**, 2485-2493

[39] Kilberg, M. S. and Haeusssinger, D. (1992) *Mammalian Amino Acid Transport: Mechanisms and Control*, Plenum Pr., New York

[40] Kim, D.W., Huang, T., Schirch, D., & V. Schirch (1996) Properties of Tetrahydropteroylpentaglutamate bound to 10-formyltetrahydrofolate dehydrogenase. *Biochem.*,**35**, 15772-15783.

[41] Klor D, Kurz J, Fuch s, Faust B, Osswald H (1996): S-adenostlhomocysteine-hydrolase from bovine kidney: enzymatic and binding properties. *Kid Blod Press Res*, **19**:100-108.

[42] Kluijtmans, L. A. J., Boers, G. H. J., Stevens, E. M. B., Renier, W. O., Kraus, J. P., Trijbels, F. J. M., van den Heuvel, L. P. W. J., and Blom, H. J. (1996) *J. Clin Invest.* **98**, 285-289

[43] Kondo, T., Dale, G.L., & Beutler, E. (1980) Glutathione transport by inside-out vesicles from human erythrocytes. *PNAS* **77**, 6359- 6362.

[44] Latta, K., & Augusteyn, R.C. (1984). The purification and properties of human lens glutathione reductase. *Experimental Eye Research.* **39**, 343-354.

[45] Lu, Y.-Z., Aiello, P.D. and Matthews, R.G. (1984). Studies on the polyglutamate specificity of thymidylate synthase from fetal pig liver. *Biochemistry*, **23**, 6870-6876.

[46] Mailliard, M. E., and Kilberg, M. S. (1990) Sodium-dependent neutral amino acid transport by human liver plasma membrane vesicles. *J. Biol. Chem.*, **265**, 14321-14326.

[47] Matthews, R.G. (1986). Methylenetetrahydrofolate reductase from pig liver. *Meth. Enzymol.*,**122**, 372-381.

[48] Mendoza-Cozatl DG, Moreno-Sanchez R. (2006) Control of glutathione and phytochelatin synthesis under cadmium stress. Pathway modeling for plants. *J Theor Biol*, **238**:919-936

[49] Morrison, P. F. and Allegra, C. J. (1989) Folate cycle kinetics in human breast cancer cells. *J. Biol. Chem.* **264**, 10562-10566.

[50] Mosharov, E., Cranford, M.R., & Banerjee, R. (2000) The quantitatively important relationship between homocysteine metabolism and glutathione synthesis by the transsulfuration pathway and its regulation by redox changes. *Biochem.* **39**, 13005-13011

[51] Nijhout, H. F., Reed, M. C., Anderson, D. A., Mattingly, J., James, S. J., Ulrich, C. M. 2006. Long-Range Allosteric Interactions between the Folate and Methionine Cycles Stabilize DNA Methylation Reaction Rate, *Epigenetics*, **1**, 81-87.

[52] Nijhout, H. F., Reed, M. C., Budu, P., and Ulrich, C. M. 2004. A Mathematical Model of the Folate Cycle. *J. Biol. Chem.* **279**, 55008-55016

[53] Ogawa, H., and Fujioka, M. (1982) *J. Biol. Chem.* **257**, 3447-3452

[54] Ookhtens M, Hobdy K, Corvasce MC, Aw TY, Kaplowitz M: Sinusoidal efflux of glutathione in the perfused rat liver. J Clin Invest 1985, 75:258-265.

[55] Ookhtens M, Maddatu T: Mechanism of changes in hepatic sinusoidal and biliary glutathione efflux with age in rats. Am J Physiol 1991, 261:G648-G656.

[56] Ookhtens M, Mittur AV, Erhart NA: Changes in plasma glutathione concentrations, turnover, and disposal in developing rats. Am J Physiol. 1994, 266:R979-R988.

[57] Ookhtens, M., & Kaplowitz, N. (1998). Role of the liver in interorgan homeostasis of glutathione and cyst(e)ine. *Seminars in Liver Disease* **18**, 313-329.

[58] Pajares, M.A., Duran, C., Corrales, F., Pliego, M.M., & Mato, J.M. (1992). Modulation of Rat Liver S-Adenosylmethionine Synthetase Activity by Glutathione. *J. Biol. Chem.* **267**, 17598-17605.

[59] Palacin, M., Estevez, R., Bertran, J., and Zorzano, A. (1998) Molecular biology of mammalian plasma membrane amino acid transporters. *Physiol. Rev.*, **78**, 969-1054.

[60] Porter, D. H., Cook, R. J., and Wagner, C. (1985) Enzymatic properties of dimethylglycine dehydrogenase and sarcosine dehydrogenase from rat liver. *Arch. Biochem. Biophys.*, **243**, 396-407.

[61] Rayl, E.A., Moroson, B.A. and Beardsley, G.P. (1996). The human purH gene product, 5-aminoimodazole-4-carboxamide ribonucleotide formyltransferase/IMP cyclohydrolase. Cloning, sequence, expression, purification, kinetic analysis, and domain mapping.*J. Biol. Chem.*,**271**, 2225-2233.

[62] Reed, M. C., Nijhout, H. F., Sparks, R., and Ulrich, C. M. (2004). A Mathematical model of the methionine cycle. *J. Theor. Biol.* **226**, 33-43.

[63] Richman PG, Meister A. (1975) Regulation of γ-glutamyl-cysteine synthetase by non-allosteric feedback inhibition by glutathione. *J. Biol. Chem.*, **250**:1422-1426.

[64] Sanchez del Pino, M. M., Corrales, F. J., and Mato, J. M. (2000) *J. Biol. Chem.* **275**, 23476-23482

[65] Savvides, S., Scheiwein, M., Bohme, C., Arteel, G., Karplus, P.A., Becker, K., & Schirmer, R.H. (2002). Crystal structure of the antioxidant enzyme glutathione reductase inactivated by peroxynitrite. *J. Biol. Chem.* **277**, 2779-2784.

[66] Schirch, V., Hopkins, S., Villar, E. and Angelaccio, S. (1985). Serine hydroxymethyltransferase from Escherichia coli: purification and properties. *J. Bacteriol.*,**163**, 1-7.

[67] Schirch, D., Villar, E., Maras, B., Barra,D. and Schirch, V. (1994) Domain structure and function of 10-formyltetrahydrofolate dehydrogenase, *J. Biol. Chem.* **269**, 24728 - 24735

[68] Schirch, V. (1997). Purification and properties of folate-dependent enzymes from rabbit liver. *Meth. Enzymol.*,**281**, 146-161.

[69] Schirch, L. & Peterson, D. (1980). Purification and properties of mitochondrial serine hydroxymethyltransferase. *J. Biol. Chem.*,**255**, 7801-7806.

[70] Seelig G, Meister A. (1995) Glutathione biosynthesis: γ-glutamylcysteine synthetase from rat kidney. *Methods in Enzymology*, **113**: 379?399.

[71] Skiba, W. E., Taylor, M. P., Wells, M. S., Mangum, J. H., and Awad, W. M. 1982. Human hepatic methionine biosynthesis. Purification and characterization of betaine:homocysteine S-methyltransferase, *J. Biol. Chem.*, **257**, 14944-14948.

[72] Seither, R. L., Trent, D. F., Mikulecky, D. C., Rape, T. J., & I. D. Goldman (1989) Folate-pool interconversions and inhibition of biosynthetic processes after exposure of L1210 leukemia cells to antifolates. *J. Biol. Chem.*, **264**, 17016-17023.

[73] Soriano-Garcia, J. F., Torras-Llort, M., Ferrer, R., and Moreto, M. (1998) Multiple pathways for L-methionine transport in brush-border membrane vesicles from chicken jejunum. *J. Physiol.*, **509.2**, 527-539.

[74] Steegborn, C., Clausen, T., Sondermann, P., Jacob, U., Worbs, M., Marinkovic, S., Huber, R., & Wahl, M. (1999). Kinetics and inhibition of recombinant human cystathione γ-lyase. *J. Biol. Chem.* **274**, 12675-12684.

[75] Stipanuk, M.H., Coloso, R.M., Garcia, R.A.G., & Banks, M.F. (1992). Cysteine concentration regulates cysteine metabolism to glutathione, sulfate, and taurine in rat hepatocytes. *J. Nutrition* **122**, 420-427.

[76] W.B., Tendler, S.J., Seither, I.D.G. and Schirch, V. (1990). Purification and properties of serine hydroxymethyltransferase and C1-tetrahydrofolate synthase from L1210 cells. *J. Biol. Chem.*,**265**,

[77] Sullivan, D. M., and Hoffman, J. L. (1983) *Biochemistry* **22**, 1636-1641.

[78] Taoka S., Ohja, S., Shan, X., Kruger, W. D., Banerjee, R. (1998) Evidence for heme-mediated redox regulation of human cystathionine beta-synthase activity. *J. Biol. Chem.*, **273**, 25179-25184.

[79] Verleysdonk, S., Martin, H., Willker, W., Leibfritz, D., and Hamprecht, B. (1999) Rapid uptake and degradation of glycine by astroglial cells in culture: synthesis and release of serine and lactate. *Glia*, **27**, 239-248.

[80] Vorontzov, I.N., Greshilov, M.M., Belousova, A.K. and Gerasimova, G.K. (1980).Mathematical description and investigation of the principles of functioning of the folic acid cycle.*Biokhimiya*, **45:**, 83-97.

[81] Yamada, K., Chen, Z., Rozen, R., and Matthews, R. G. (2001) *PNAS* **98**, 14853-14858

[82] Wagner, C. (1995) Biochemical role of folate in cellular metabolism, in *Folate in Health and Disease*(Bailey, L. B., ed.),pp. 23-42, Marcel Dekker, New York.

[83] Wu, G., Fang, Y., Yang, S., Lupton, J., & Turner, N. (2004). Glutathione metabolism and its implications for health. *J. Nutrition* **134** , 489-492.

[84] Yeo, E.-J., and Wagner, C. (1992) *J. Biol. Chem.* **267**, 24669-24674
